# Socio-environmental and psychosocial predictors of smoking susceptibility among adolescents with contrasting socio-cultural characteristics: a comparative analysis

**DOI:** 10.1186/s12889-021-12351-x

**Published:** 2021-12-09

**Authors:** Christopher Tate, Rajnish Kumar, Jennifer M. Murray, Sharon Sanchez-Franco, Shannon C. Montgomery, Felipe Montes, Laura Dunne, Olga L. Sarmiento, Frank Kee, Ruth F. Hunter

**Affiliations:** 1 Centre for Public Health, Institute of Clinical Sciences Block B, Queen’s University Belfast, Royal Victoria Hospital, Grosvenor Road, Belfast, BT12 6BA UK; 2 Queen’s Management School, Queen’s University Belfast, Riddel Hall, 185 Stranmillis Road, Belfast, BT9 5EE UK; 3 Department of Public Health, School of Medicine, Universidad de Los Andes, Carrera 1 No 18 A – 10, Bloque Q Piso 8, Bogotá, Postal Code: 57 Colombia; 4 Department of Family and Child Sciences, Florida State University, Sandels Building, 675 W Call St, Tallahassee, FL 32304 USA; 5 Department of Industrial Engineering, Social and Health Complexity Center, Universidad de Los Andes, Bogotá, Colombia; 6 Centre for Evidence and Social Innovation, School of Social Sciences, Education and Social Work, Queen’s University Belfast, 2-8 Fitzwilliam Street, Belfast, BT9 6AW UK

**Keywords:** Adolescent, Smoking, Social norms, Risk factors, Psychosocial, Cognitions

## Abstract

**Background:**

Despite a steady decline in adolescent smoking globally, it remains a prevalent risk factor for non-communicable disease. Previous research points to differences in socio-environmental and psychosocial risk factors for smoking and how they vary across different settings with disparate social and cultural characteristics. As a result, smoking rates have remained disproportionately higher in some settings while decreasing in others. This study explored the socio-environmental and psychosocial risk factors for smoking susceptibility in a high-income and upper-middle income setting.

**Methods:**

Cross-sectional data were obtained from 1,573 male and female adolescents aged 11-15 years who completed self-administered questionnaires in schools in Northern Ireland and Bogotá, Colombia. Using logistic regression analysis, we examined how socio-environmental and psychosocial predictors of smoking susceptibility compared across the two countries.

**Results:**

In Northern Ireland, reduced odds of smoking susceptibility were significantly associated with less family smoking (OR: 0.64, 95% CI: 0.41-1.00); having access to information about smoking in school (OR: 0.75, 95% CI: 0.59-0.96); negative attitudes towards smoking (OR: 0.35, 95% CI: 0.23-0.51); higher levels of openness (OR: 0.59, 95% CI: 0.50-0.69); and higher levels of self-reported wellbeing (OR: 0.57, 95% CI: 0.44-0.74). Increased odds of smoking susceptibility were associated with reporting less smoking of a mother (OR: 1.37, 95% CI: 1.06-1.76); higher levels of extraversion (OR: 1.40, 95% CI: 1.04-1.90); and receiving pocket money (OR: 1.20, 95% CI: 1.06-1.37). In Bogotá, reduced odds of smoking susceptibility were significantly associated with reporting less smoking among friends (OR: 0.86, 95% CI: 0.76-0.98); higher levels of self-efficacy (OR: 0.58, 95% CI: 0.40-0.83); greater perceived behavioural control to quit smoking (OR: 0.71, 95% CI: 0.56-0.90); and lower levels of truancy (OR: 0.69, 95% CI: 0.52-0.92). In Bogotá, no factors were associated with increased odds of smoking susceptibility in the final model.

**Conclusions:**

The findings illustrate that there were differences in predictors of adolescent smoking susceptibility across the two settings. By using a comparative approach we demonstrate that smoking interventions and policies must be sensitive to the cultural and normative context within which they are implemented.

**Supplementary Information:**

The online version contains supplementary material available at 10.1186/s12889-021-12351-x.

## Background

Smoking is an important modifiable risk factor for non-communicable diseases (NCD) in both high-income countries as well as low- and middle-income countries (LMICs), not least because of its role as a precursor and accessory to other risk behaviours among younger populations [1–3]. In Northern Ireland, a high-income constituent country of the United Kingdom, it is estimated that current cigarette consumption amongst adolescents aged 11-16 years is 4% [4]. By comparison, in Colombia, an upper-middle income country, data suggests 8.1% of adolescents aged 12-18 years smoke, and in the capital city, Bogotá, estimates place this figure at 13.1% [5].

Smoking susceptibility (the absence of a firm commitment *not* to smoke) is inherently linked to experimentation which predisposes adolescents to subsequent cigarette smoking [6, 7]. The importance of susceptibility to smoking within the developmental context of adolescence is significant given the role of behavioural intentions in predicting future behaviour [8]. Studies have harnessed smoking susceptibility both as a predictor of future smoking [9, 10] as well as an outcome of various social and behavioural risk factors [11, 12]. As such, there is value in understanding the factors that predict smoking susceptibility due to the implications for the subsequent formation of smoking habits that can extend into adulthood.

Various studies point to the role of social norms (both descriptive and injunctive) in adolescent smoking. Descriptive norms are the perception of what behaviours are performed by others [13]. Injunctive norms correspond to the perceived pressures to conform to a behaviour to avoid social sanctions [14]. The perceived prevalence and perceived acceptability of smoking among peers and family members have been shown to predict adolescent smoking [15–18].

Studies have also investigated the proximal social factors in family and peer contexts [19, 20], as well as distal (upstream) influences emerging from cultural spheres of influence such as exposure to smoking-related media content [21]. For example, it has been shown that adolescents who report higher levels of exposure to smoking in movies are at a greater risk of commencing smoking [22, 23]. However, the socio-environmental factors conducive to smoking are not universal across all contexts, therefore, it cannot be assumed that smoking intentions are regulated entirely by external factors.

Self-efficacy, the belief an individual holds about their ability to exercise control over their own actions and associated outcomes [24], is well established as a determinant of adolescent smoking [25–27]. For example, refusal self-efficacy has been shown to predict smoking among adolescent boys in a study of Chinese youth [28]. Further, another study [29] found that self-efficacy mediated the association between smoking behaviour and social influence.

Cognitive constructs, such as perceived behavioural control (PBC) and attitude towards smoking, have also been shown to be associated with adolescent’s intentions to smoke [30]. Furthermore, adolescents knowledge of the potential side effects and perceived benefits of smoking can alter their intentions. For example, adolescent smokers report that they perceive themselves as being less likely to become addicted [31], less likely to suffer negative health-related side effects [32], and perceive greater social benefits of smoking [33].

Personality factors were examined in an earlier study [34] that used the five personality dimensions (or “Big Five”) [35]. It was reported that students who scored higher on the extraversion dimension and lower on the emotional stability dimension were consistently more likely to smoke. This finding was reiterated in another study [36] that found adolescents who exhibited more extraverted behavioural traits were at higher risk of smoking, whereas greater emotional stability was protective.

Research indicates that other psychosocial factors such as emotional well-being [37] and life-satisfaction [38] are also protective factors against adolescent smoking. Conversely, depressive symptoms [39], low self-esteem [40], emotional or behavioural problems [41], low life satisfaction [42], and high levels of anxiety [43] are reported to be predictive of adolescent smoking initiation.

This study adds a cross-cultural perspective to the wealth of existing evidence that already highlights the importance of both socio-environmental and individual-level factors that contribute to smoking among adolescents. Importantly, by providing a direct comparison between settings characterised by distinct socio-cultural and normative characteristics, cross-cultural research offers insight into potential ways of optimising intervention policies and preventative strategies to accommodate the variability of risk factors for smoking across settings. Using data obtained from adolescents in Northern Ireland and Bogotá, Colombia, the objective of this study was to compare and contrast socio-environmental and individual-level factors associated with smoking susceptibility in a high-income setting and upper-middle income setting.

## Methods

### Study sample

Study participants were a cross-sectional sample from the first wave of data collection of the Mechanisms of Networks and Norms Influence on Smoking in Schools (MECHANISMS) study. The MECHANISMS study was a school-based study designed to further understanding of social norms based mechanisms of action related to smoking in high- and middle-income settings. Baseline data collection took place in Northern Ireland and Bogotá, Colombia before students participated in school-based smoking prevention interventions.

Cross-sectional data were collected from 1,573 students aged 11-15 years in a post-primary educational setting in schools in Northern Ireland, UK (*n* = 7) and Bogotá (*n* = 8). In Northern Ireland, the sample of schools served urban and rural catchments, and maximum variation sampling was used to ensure there was an adequate balance of schools with high and low proportions of pupils eligible for free school meals. Eight public schools in Bogotá were identified using a comparable maximum variation sampling approach. Sampling of schools in Bogotá was performed in three steps: first, 40 private and public schools were identified based on health risks by the Education and Health Departments of Bogotá; second, 13 schools were shortlisted for inclusion in the study if they were situated in an urban area, were mixed-gender, and had an enrolment of 90-150 students in year 7; third, six schools accepted the invitation to participate in the study and were subsequently selected.

Participants (50% female) completed a baseline self-report survey measuring a range of variables pertaining to socio-environmental risk factors for smoking, smoking-related cognitions, and psychosocial traits. The self-administered questionnaire was based conceptually on key variables from the Theory of Planned Behaviour [44], namely attitudes, subjective norms, and PBC. Theory of Planned Behaviour constructs were supplemented with additional measures identified in the literature as having a significant bearing on adolescent smoking intentions. A description of the scales used is included in the Appendix. The questionnaire items are shown in Table 1 in the Appendix.

### Ethical considerations

All pupils were required to complete consent forms indicating whether they agree or decline to participate. A parental opt-out procedure was used whereby parents/guardians who did not wish their child to take part were asked to return completed opt-out forms. Pupils who consented to participate were asked to complete a baseline assessment. Ethical approval was obtained prior to the first wave of data collection. Ethical approval for this study was granted by the Queen's University Belfast, School of Medicine, Dentistry and Biomedical Sciences Ethics Committee in September 2018, and Research Committee of the Universidad de Los Andes, Bogotá in July 2018 (see the study protocol [45] for full details of the study design).

### Smoking susceptibility

Susceptibility to smoking was defined as the absence of a firm commitment not to smoke [6]. Participants were classified as susceptible or not based on three items measuring intentions to smoke:

1. Do you think you will try a cigarette soon?
2. If one of your best friends were to offer you cigarette, would you smoke it?
3. If you don’t currently smoke, do you intend to take up smoking in the next 6 months?

The student was coded as not susceptible if they answered ‘No’ (from three choices), ‘Definitely not’ (from five choices), and ‘Definitely not’ (from six choices) respectively to these questions. The student was coded as susceptible with any other set of responses.

### Socio-demographic factors

Socio-demographic data collected in the baseline survey included gender, age, socioeconomic level based on country-specific measures, ethnicity and family structure. Student and school deprivation ranks were obtained for Northern Ireland from Northern Ireland and Statistics Research Agency data [46]. Student and school socioeconomic level indexes for Bogotá were obtained from data published by the Colombian Institute for the Evaluation of Education [47].

### Socio-environmental factors

Injunctive norms were assessed with seven subscales and descriptive norms were assessed with eight subscales [48]. Exposure to advertising in the media was assessed with eight items [49]. Exposure to tobacco advertising in shops was measured using four items [50]. School smoking information was assessed with a single item asking, “Do you think your school has given you enough information on smoking?”.

### Smoking-related cognitions

Self-efficacy was assessed using three subscales: (i) emotional; (ii) friends; and (iii) opportunity (Cronbach’s α: 0.981) [51, 52]. PBC was assessed with two items that assessed PBC to quit and PBC to avoid smoking [30]. Perceived risks and benefits of tobacco-use were assessed using two separate scales: perceived risks (13 items; α = 0.864); and perceived benefits (five items; α = 0.774) [31]. Attitudes towards smoking were assessed a 12-item scale [53] (α = 0.787). Knowledge of health effects of smoking was assessed with the 6-item scale [48].

### Psychosocial characteristics and personality traits

Need to belong was measured using 10 items (α = 0.813) [54, 55]. Fear of negative evaluation was assessed with 12 items (α = 0.894) [55–57]. The Prosocial Behaviour score was derived from 5 items (α = 0.733) [55, 58]. We assessed personality traits [35] by using the Big Five Personality Trait Short Questionnaire (BFPTSQ). Each dimension was measured using a 10-item subscale: openness (α = 0.798); extraversion (α = 0.776); agreeableness (α = 0.700); conscientiousness (α = 0.700); and emotional stability (α = 0.745). In Northern Ireland, we used the questionnaire validated for English-speaking adolescents [59]. In Bogotá, we used the questionnaire validated for Spanish-speaking adults [60]. Self-perceived wellbeing was measured using five items (α = 0.821) [61]. Truancy, and access to and disposal of pocket money were assessed using questions adapted from an earlier study [50].

### Statistical analyses

The analysis used univariate and multivariate logistic regression modelling adjusted for clustering at country and school level to test the probability of a participant being either susceptible or not susceptible to smoking based on the variables outlined above. Three independent regressions were performed on: the whole sample (*n* = 1,573); the Northern Ireland sub-sample (*n* = 701); and the Bogotá sub-sample (*n* = 872). To account for differences in scales used to measure the independent variables a new scale was calculated using z-scores. Interaction analysis was used to determine if there was a statistically significant different in predictors of susceptibility according to country.

**Fig. 1:**
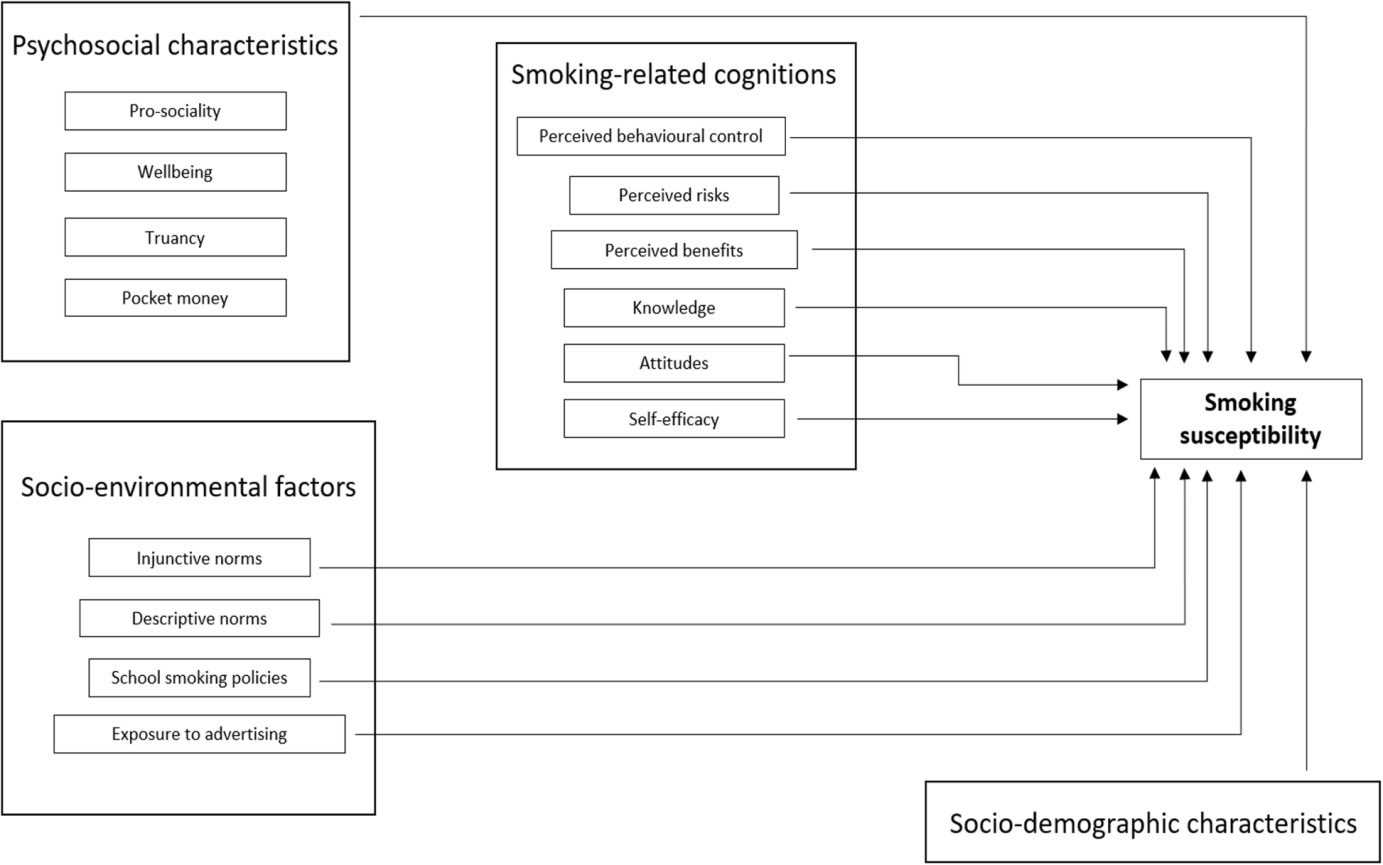
Conceptual framework of socio-environmental and psychosocial factors contributing to smoking susceptibility

A Hosmer-Lemeshow goodness-of-fit test and receiver operating characteristic analysis were used to evaluate the predictive accuracy of the final model. The conceptual framework that guided the analysis is presented in Fig. 1. All statistical analyses were conducted using Stata 16.1 (StataCorp, 2019, Stata Statistical Software: Release 16. College Station, TX: StataCorp LLC.).

A Pearson’s product-moment correlation matrix was used to assess if there was any potential associations among the predictor variables. The strength of the association between independent variables was assessed using Cohen’s [62] guidelines for interpreting the magnitude of correlation coefficients. As an additional tool to check for multicollinearity, variation inflation factors (VIF) (VIF ≥ 10 suggests that variables are measuring similar constructs) and tolerance scores were analysed post-hoc [63].

## Results

Table 1 shows the socio-demographic characteristics of the student sample. Both samples demonstrate similar socioeconomic characteristics, with the majority of students being categorised in low – middle socioeconomic ranking scales. A smaller proportion of the students in the Bogotá sample live with both parents (55%) when compared to the Northern Ireland sample (80%).

**Table 1:** Sample socio-demographic characteristics

Sample Characteristics^a^	Total (*n* = 1,573)		Northern Ireland (*n* = 701)		Bogotá (*n* = 872)		Chi-square (*χ*^2^) *p*-value
**Demographics**							
Female	786	(50%)	355	(51%)	431	(49%)	0.117
Age, years							0.000
11	27	(2%)	1	(<1%)	26	(3%)	
12	598	(38%)	279	(40%)	319	(37%)	
13	722	(46%)	414	(59%)	308	(35%)	
14	151	(10%)	7	(1%)	144	(17%)	
15 or more	75	(5%)	0	(0%)	75	(9%)	
Ethnicity							0.000
Non-ethnic minority	1,401	(89%)	648	(93%)	753	(86%)	
Ethnic minority	170	(11%)	51	(7%)	119	(14%)	
**Socioeconomic measures**							
Student Deprivation Rank^b^							
Low			275	(39%)			
Middle			216	(31%)			
High			137	(20%)			
School Deprivation Rank^b^							
Low			364	(52%)			
Middle			231	(33%)			
High			106	(15%)			
Student Socioeconomic Level^c^							
Lowest					7	(1%)	
Low					240	(28%)	
Middle – Low					313	(36%)	
Middle					242	(28%)	
Middle – High					50	(6%)	
High					2	(<1%)	
School Socioeconomic Level^d^							
Middle – Low					544	(62%)	
Middle – High					328	(38%)	
**Family structure**							0.000
Single parent	465	(30%)	126	(18%)	339	(39%)	
Both parents	1039	(66%)	557	(80%)	482	(55%)	
Live with other adult	67	(4%)	16	(2%)	51	(6%)	

^a^Variable distributions are reported as n (%) unless otherwise stated

^b^Northern Ireland only. Low (0-300), Middle (301-600), High (601-890). Northern Ireland Multiple Deprivation Measure rank derived from NISRA data

^c^Bogotá, Colombia only. Socioeconomic level index of individual students according to the Departamento Administrativo Nacional de Estadística (DANE; "National Administrative Department of Statistics")

^d^Socioeconomic level Index of schools according to the Instituto Colombiano para el Fomento de la Educación Superior ("Colombian Institute for the Promotion of Higher Education")

The smoking characteristics of the sample are shown in Table 2. A smaller proportion of students in the Bogotá cohort reported having never smoked (80%) compared to Northern Ireland (85%). A larger proportion of students in Bogotá (43%) were classified as susceptible compared to the Northern Ireland cohort (31%).

**Table 2:** Sample smoking behaviour and intentions characteristics

Sample characteristics^a^	Total (*n* = 1,573)		Northern Ireland (*n* = 701)		Bogotá (*n* = 872)		*χ*^2^ p-value
Smoking behaviour							0.004
Current smoker	58	(4%)	27	(4%)	31	(4%)	
Previous smoker	224	(14%)	77	(11%)	147	(17%)	
Never smoker	1291	(82%)	597	(85%)	694	(80%)	
**Smoking Susceptibility**							
Try a cigarette soon							0.000
Yes	34	(2%)	17	(2%)	17	(2%)	
Don’t know	375	(24%)	107	(15%)	268	(31%)	
No	1163	(74%)	576	(82%)	587	(67%)	
Missing Values	1	(<1%)	1	(<1%)	0	(0%)	
Best friend offered cigarette							0.000
Definitely yes	6	(<1%)	4	(1%)	2	(<1%)	
Probably yes	53	(3%)	26	(4%)	27	(3%)	
Not sure	184	(12%)	58	(8%)	126	(14%)	
Probably not	151	(10%)	84	(12%)	67	(8%)	
Definitely not	1177	(75%)	527	(75%)	650	(75%)	
Smoke in next 6 months							0.000
Current smoker	49	(3%)	10	(1%)	39	(4%)	
Definitely start smoking	3	(<1%)	1	(<1%)	2	(<1%)	
Probably start smoking	8	(1%)	0	(0%)	8	(1%)	
Don’t know	141	(9%)	49	(7%)	92	(11%)	
Probably not	104	(7%)	45	(6%)	59	(7%)	
Definitely not	1264	(81%)	592	(84%)	672	(77%)	
**Susceptible to smoking**							0.000
Yes	587	(37%)	215	(31%)	372	(43%)	
No	985	(63%)	485	(69%)	500	(57%)	

^a^Variable distributions are reported as n (%) unless otherwise stated

The unadjusted odds ratios (OR), odds ratios adjusted for sociodemographic characteristics, and multivariate-adjusted odds ratios are reported in Tables 2, 3, and 4 respectively in the Appendix. Corresponding 95% confidence intervals (CI) and *p*-values are also reported.

### Factors associated with smoking susceptibility in both countries

In the unadjusted model (Table 2 in the Appendix), all socio-environmental factors demonstrated statistically significant associations with smoking susceptibility (p < 0.05). Self-efficacy, PBC to avoid smoking, and perceived risks of smoking were significantly negatively associated with the odds of being susceptible in the unadjusted model. Conversely, perceived benefits was positively associated with the odds of being susceptible. Students who held more negative attitudes towards smoking and had greater knowledge of the health effects of smoking were less likely to be susceptible. A higher score on each of the Big Five personality dimensions significantly predicted a reduced likelihood of being susceptible.

Students from Bogotá were statistically more likely to be susceptible to smoking, as were those who were older.

After adjusting for sociodemographic factors (Table 3 in the Appendix), the odds ratios for smoking susceptibility remained lower for adolescents who reported fewer injunctive norms favourable to smoking and fewer descriptive smoking norms. The odds of being susceptible remained higher for students reporting more frequent exposure to smoking in media content and smoking advertising in shops. Higher levels on the fear of negative evaluation scale significantly increased the odds of being susceptible, after adjusting for sociodemographic factors. Only PBC to quit smoking, need to belong, and receiving pocket money did not significantly predict the odds of being susceptible in this model.

The results of the multivariate-adjusted analysis (Table 4 in the Appendix) differed from those of the univariate analysis in a number of ways. After adjusting for all variables, descriptive norms pertaining to sister(s) smoking (OR: 1.12, 95% CI: 1.06 - 1.18) significantly predicted the odds of being susceptible, as did injunctive norms for important people (OR: 0.86, 95% CI: 0.76 - 0.97), father (OR: 1.15, 95% CI: 1.00 - 1.33), sister(s) (OR: 0.94, 95% CI: 0.91 - 0.98), and friends (OR: 0.79, 95% CI: 0.76 - 0.82). Additionally, cigarette advertising in shops remained a significant socio-environmental predictor of smoking susceptibility (OR: 1.07, 95% CI: 1.03 - 1.11). Greater self-efficacy (OR: 0.59, 95% CI: 0.53 - 0.65), perceiving more risks associated with smoking (OR: 0.86, 95% CI: 0.85 - 0.86), and more negative attitudes towards smoking (OR: 0.62, 95% CI: 0.47 - 0.80) significantly reduced the odds of being susceptible in the fully adjusted model.

Among the psychosocial factors, scoring higher on the need to belong scale positively predicted the odds of smoking susceptibility (OR: 1.09, 95% CI: 1.01 - 1.16). In contrast, a higher score on the prosociality scale (OR: 0.95, 95% CI: 0.95 - 0.96) and conscientiousness scale (OR: 0.92, 95% CI: 0.90 - 0.94) significantly reduced the odds of being susceptible as well as lower rates of truancy (OR: 0.72, 95% CI: 0.67 - 0.78). Students who reported that they were restricted with regards to how they spent pocket money were also less likely to be susceptible (OR: 0.92, 95% CI: 0.89 - 0.94).

Age (OR: 1.04, 95% CI: 1.03 - 1.04) and country (OR: 1.50, 95% CI: 1.04 - 2.15) were the only sociodemographic factors that significantly predicted the odds of being susceptible in the fully adjusted model.

### Factors associated with smoking susceptibility across countries

In the univariate model (Table 2 in the Appendix), examining the results from the Northern Ireland and Bogotá cohorts separately showed minimal deviation from the results obtained with the whole sample. All socio-environmental factors significantly predicted the odds of being susceptible in Northern Ireland. In Bogotá, injunctive norms from the family context (excluding mother) were not significant, nor was access to information about smoking in school. The demographic factors age, socioeconomic status and school socioeconomic status were significant in Bogotá, while no sociodemographic factors were significant in Northern Ireland.

After adjusting for socio-demographic factors (Table 3 in the Appendix), all socio-environmental factors significantly predicted smoking susceptibility in Northern Ireland, with the exception of father injunctive norms. In Bogotá, two types of injunctive norm (father and brother), sister(s) descriptive norms, and school smoking information were non-significant.

In the fully adjusted model (Table 4 in the Appendix), descriptive norms from two sources (mother (OR: 1.37, 95% CI: 1.06 - 1.76) and family (OR: 0.64, 95% CI: 0.41 - 1.00)) and school smoking information (OR: 0.75, 95% CI: 0.59 - 0.96) significantly predicted the odds of being susceptible in Northern Ireland. By comparison, friend descriptive norms (OR: 0.86, 95% CI: 0.76 - 0.98) was the only significant socio-environmental variable in Bogotá. Interaction analysis confirmed that school smoking information differed significantly across the two settings (OR: 0.75, *p* = 0.024 in Northern Ireland compared to OR: 1.09, *p* = 0.313 in Bogotá).

There was some variation in smoking-related cognitions as predictors of smoking susceptibility across the two countries. In Northern Ireland, the univariate analysis showed self-efficacy, perceived risks of smoking, perceived benefits of smoking, PBC to avoid smoking, and attitudes towards smoking significantly predicted susceptibility. In Bogotá, self-efficacy, perceived risks of smoking, PBC to avoid smoking, attitudes towards smoking, and knowledge of the health effects significantly predicted susceptibility.

Adjusting for sociodemographic factors produced no significant change in the estimates for smoking-related cognitions in either country.

In the fully adjusted model, attitude (OR: 0.35, 95% CI: 0.23 - 0.51) maintained a significant association with smoking susceptibility in Northern Ireland. In Bogotá, self-efficacy (OR: 0.58, 95% CI: 0.40 - 0.83) and PBC to quit smoking (OR: 0.71, 95% CI: 0.56 - 0.90) significantly predicted susceptibility. In this model, attitude toward smoking was the only smoking-related cognition that differed significantly between the two countries (OR: 0.35, *p* = 0.000 in Northern Ireland compared to OR: 0.68, *p* = 0.100 in Bogotá).

Of the Big Five personality dimensions, only extraversion was statistically non-significant in Northern Ireland in the univariate model, while higher scores on the remaining Big Five subscales were associated with lower odds of being susceptible in both countries. Students who reported higher levels of wellbeing in Northern Ireland and Bogotá were less likely to be susceptible. Similarly, students who reported lower levels of truancy had lower odds of being susceptible to smoking in both Northern Ireland and Bogotá in the univariate model.

After adjusting for sociodemographic factors, fear of negative evaluation was no longer a significant predictor in Northern Ireland. Adjusting for sociodemographic factors produced no change in the variables that predicted smoking susceptibility in Bogotá.

In the multivariate-adjusted model, openness (OR: 0.59, 95% CI: 0.50 - 0.69), extraversion (OR: 1.40, 95% CI: 1.04 - 1.90), wellbeing (OR: 0.57, 95% CI: 0.44 - 0.74), and receiving pocket money (OR: 1.20, 96% CI: 1.06 - 1.37) demonstrated a significant association with smoking susceptibility in Northern Ireland, while truancy (OR: 0.69, 95% CI: 0.52 - 0.92) was the only psychosocial variable that significantly predicted susceptibility in Bogotá. OR estimates for agreeableness, wellbeing and receiving pocket money differed significantly across countries in the final model.

As shown in the Pearson’s product-moment correlation matrix for both countries (Table 5 in the Appendix), a high proportion of the independent variables were correlated, however the strength of the association was small for most. Self-efficacy was positively correlated with both injunctive and descriptive norms (p < 0.05), however, the strength of the association was small for most subscales (r < .3). The VIF and tolerance scores for the independent variables included in the final analysis for both countries indicated that no variables exhibited signs of meaningful collinearity in our analysis (Table 6 in the Appendix).

## Discussion

Previous research has shown that a disproportionate number of those aged 15 years and over who smoke (approximately 80%) live in LMICs [64]. This is concerning given the role of initiation during early adolescence as a risk factor for subsequent smoking [65]. This study investigated differences between the socio-environmental and individual-level risk factors for smoking susceptibility in a high-income country (Northern Ireland) and upper-middle income country (Bogotá, Colombia). Findings from logistic regression analyses illustrated differences between the two settings regarding descriptive norms, smoking-related cognitions, and psychosocial traits. In Northern Ireland, adolescents who reported that fewer family members smoked were less likely to be susceptible to smoking. In Bogotá, reporting that fewer friends smoked reduced the odds of being susceptible. Reduced odds of being susceptible to smoking were significantly associated with negative attitudes towards smoking in Northern Ireland, while higher levels of self-efficacy and PBC to quit were associated with reduced odds in Bogotá. When psychosocial traits were examined, higher levels of openness and self-reported wellbeing significantly reduced the odds of being susceptible in Northern Ireland. Conversely, higher levels of extraversion increased the odds of being susceptible in Northern Ireland. In Bogotá, students who did not skip school were less likely to be classified as susceptible to future smoking.

Descriptive and injunctive norms are reported in the literature as risk factors for smoking among adolescents [66–68]. We did find evidence of a significant association between injunctive norms and smoking susceptibility in both countries after adjusting for sociodemographic factors, however, this relationship was not significant in our final model. In the composite sample, injunctive norms favourable to smoking from important people, father, sister(s), and friends were significant in the final model.

We did find support for the role of descriptive norms in predicting smoking susceptibility in the final model. Students in Bogotá who reported less smoking among friends were less likely to be susceptible. In addition, fewer descriptive smoking norms among family reduced the odds of being susceptible in Northern Ireland. Interestingly, reporting less smoking by a mother increased the odds of being susceptible in Northern Ireland in the final model.

In agreement with other studies pointing to a link between pro-smoking messages in media content and an elevated risk of susceptibility to smoking [69, 70], we found a significant correlation between exposure to smoking-related media content and smoking susceptibility in both countries after adjusting for sociodemographic factors. However, this association was no longer significant in the final model. This is in contrast to another study of LMIC settings which found that adolescents who were exposed to smoking in electronic media were more likely to be smokers [71]. Similar to the results of other studies [72, 73], we found exposure to cigarette advertising in shops was a significant predictor of susceptibility in both countries after adjusting for sociodemographic factors. In the final model, this finding was limited to the composite sample.

Consistent with previous research [74] that showed refusal self-efficacy was protective against smoking initiation, we found a statistically significant association between refusal self-efficacy and smoking susceptibility. Additionally, our results concur with the findings of an earlier study [31] that found students who perceived greater risks associated with smoking were at less risk for future initiation. However, when the data were disaggregated by country in the final model, the association was no longer significant. In line with previous findings [33], we found a statistically significant link between perceived benefits of smoking and susceptibility after adjusting for sociodemographic factors in Northern Ireland. Further, our results confirmed a significant correlation between knowledge of the harmful effects of smoking and susceptibility in Bogotá. However, in the final model, knowledge was not a significant predictor of susceptibility in either country. This echoes the findings from an earlier study [75], but contrasts with others [16] who found that lack of knowledge about the harms of smoking predicted ever-smoking.

Of the Big Five Personality factors, only two (openness and extraversion) significantly predicted the odds of being susceptible in Northern Ireland in the final model. In Northern Ireland, our results show that students who were more receptive to new ideas or experiences and expressed a greater tendency to be curious were less likely to be susceptible. This finding coincides with an earlier study [76] that reported lower levels of openness were associated with intentions to smoke. The current analysis also showed that more extraverted students were at greater risk of being susceptible to smoking, adding to the results of two earlier studies [34, 36].

Self-reported wellbeing was a protective factor against smoking susceptibility in both countries after adjusting for sociodemographic factors, and in Northern Ireland in the final model. While studies have shown that a direct inverse relationship exists between life-satisfaction and smoking behaviour [38, 77], our final model did not demonstrate this in Bogotá. We did, however, find a significant positive association between truancy and susceptibility in Bogotá, reinforcing the findings from previous studies [78, 79].

The results reaffirm that refusal self-efficacy and adolescents’ attitudes towards smoking are important targets for prevention interventions in both LMICs and high-income countries. Interventions directed at younger populations should focus on mitigating pro-smoking social influences such as exposure to tobacco advertising by providing appropriate education about the negative side-effects of smoking and equipping adolescents with the necessary skills to refuse cigarettes. Moreover, the results highlight the differences in risk factors for smoking across the two countries, further emphasising the need for smoking prevention policies to be sensitive to the normative and cultural context within which they are implemented.

### Strengths and limitations

There were several limitations of this study. Firstly, responses from the survey may be subject to social desirability bias which is not uncommon for self-report surveys [80]. As a result, students may have underreported smoking behaviors [81]. However, studies have shown self-reports of smoking behaviour are reliable [82]. Secondly, estimates of the smoking of friends and family members would potentially be subject to individual biases or “pluralistic ignorance” [83]. Thirdly, students who did not participate were potentially more likely to be smokers who did not want to report their behaviour [84].

The findings from this study may not generalise to other populations due to cultural and social factors unique to the two settings. However, the study used robust maximum variation sampling to ensure there was sufficient heterogeneity between schools in both countries serving urban and rural areas. To ensure the validity of student’s responses participants were assured their responses would not be shared with other students or teachers. Students were also assigned a unique identification number to anonymise their responses.

In the fully adjusted model for both countries, the Hosmer-Lemeshow goodness of fit test yielded a p-value of .1752 indicating that the model fit the data well. This was also true for the Bogotá (*p* = 0.8623) model but not the Northern Ireland model (*p* = 0.0112). Receiver operating characteristic (ROC) analysis was performed to evaluate the discriminative accuracy of our final models which included data from both countries. This is demonstrated by the ROC curve which was plotted to visually illustrate the concordance between model estimates of susceptibility and observed susceptibility to smoking (referred to as the C-statistic; Figures 1, 2, and 3 in the Appendix). The C-statistics were .838, .903, and .828 for the dual-country model, Northern Ireland model, and Bogotá model respectively, indicating that the models achieved acceptable levels of discrimination [85].

## Conclusions

In sum, the results of the present study suggest there are differences in socio-environmental and psychosocial correlates of smoking susceptibility in the high-income setting of Northern Ireland and upper-middle income setting of Bogotá. For example, reporting fewer descriptive smoking norms among friends was protective against smoking susceptibility in Bogotá, but not Northern Ireland. Students who reported that their school provided information about smoking were less at risk in Northern Ireland, but not in Bogotá. Greater self-efficacy was significantly associated with a lower risk of smoking in Bogotá, highlighting the importance of self-efficacy as a mitigating factor against socio-environmental influences, such as being offered a cigarette by a friend. Exploration of how group identities that prescribe behavioural norms in each country may provide potential insights into the mechanisms underlying the formation of these behaviours within the intragroup context and the impact this has on an individual’s self-efficacy [86].

We affirm that the cultural, normative and social factors unique to each setting provided a good basis for comparison of risk factors across the socioecological spectrum.

## Supplementary Information

**Additional file 1.**

## Data Availability

The datasets used and/or analysed during the current study are available from the corresponding author on reasonable request.

## Abbreviations

LMIC: Low- and middle-income country
NCD: Non-communicable disease

## References

[1] Chen X, Unger JB, Palmer P, Weiner MD, Johnson CA, Wong MM (2002). Prior cigarette smoking initiation predicting current alcohol use: evidence for a gateway drug effect among California adolescents from eleven ethnic groups. Addict Behav..

[2] Siqueira LM, Brook JS (2003). Tobacco use as a predictor of illicit drug use and drug-related problems in Colombian youth. J Adolesc Heal..

[3] Oppong Asante K, Kugbey N (2019). Alcohol use by school-going adolescents in Ghana: Prevalence and correlates. Ment Heal Prev..

[4] Foster C, Scarlett M, Stewart B (2020). Young persons’ behaviour and attitude survey 2019 - substance use [Internet].

[5] Ministry of Justice and Law, Ministry of National Education M of H and SP. National Study of psychoactive substance use in school population Colombia [Internet]. 2016 [cited 2020 Oct 8]. Available from: https://www.unodc.org/documents/colombia/2018/Junio/CO03142016_estudio_consumo_escolares_2016.pdf.

[6] Pierce JP, Choi WS, Gilpin EA, Farkas AJ, Merritt RK (1996). Validation of susceptibility as a predictor of which adolescents take up smoking in the United States. Heal Psychol..

[7] Jackson C (1998). Cognitive susceptibility to smoking and initiation of smoking during childhood: a longitudinal study. Prev Med..

[8] Ajzen I, Ajzen I, Albarracin D, Hornik R (2007). Predicting and changing behavior: a reasoned action approach. Prediction and change of health behavior: applying the reasoned action approach.

[9] Dube SR, Arrazola RA, Lee J, Engstrom M, Malarcher A (2013). Pro-tobacco influences and susceptibility to smoking cigarettes among middle and high school students--United States, 2011. J Adolesc Heal..

[10] Lechner WV, Murphy CM, Colby SM, Janssen T, Rogers ML, Jackson KM (2018). Cognitive risk factors of electronic and combustible cigarette use in adolescents. Addict Behav..

[11] Owotomo O, Maslowsky J (2018). Adolescent smoking susceptibility in the current tobacco context: 2014-2016. Am J Health Behav..

[12] Kamke K, Sabado-Liwag M, Rodriquez EJ, Pérez-Stable EJ, El-Toukhy S (2020). Adolescent smoking susceptibility: gender-stratified racial and ethnic differences, 1999-2018. Am J Prev Med..

[13] Cialdini RB, Kallgren CA, Reno RR, Zanna MP (1991). A focus theory of normative conduct: a theoretical refinement and reevaluation of the role of norms in human behavior. Advances in experimental social psychology.

[14] Cialdini RB, Reno RR, Kallgren CA (1990). A focus theory of normative conduct: recycling the concept of norms to reduce littering in public places. J Pers Soc Psychol..

[15] Chen J, Ho SY, Wang MP, Lam TH (2018). Parental smoking, rejection of parental smoking, and smoking susceptibility and behaviors in Hong Kong adolescents. Addict Behav..

[16] Ho SY, Chen J, Leung LT, Mok HY, Wang L, Wang MP (2019). Adolescent smoking in Hong Kong: prevalence, psychosocial correlates, and prevention. J Adolesc Heal..

[17] Vitória P, Pereira SE, Muinos G, De Vries H, Lima ML (2020). Parents modelling, peer influence and peer selection impact on adolescent smoking behavior: a longitudinal study in two age cohorts. Addict Behav..

[18] Zaleski AC, Aloise-Young PA (2013). Using peer injunctive norms to predict early adolescent cigarette smoking intentions. J Appl Soc Psychol..

[19] Mercken L, Candel M, Willems P, de Vries H (2007). Disentangling social selection and social influence effects on adolescent smoking: the importance of reciprocity in friendships. Addiction..

[20] Wang Y, Tian L, Huebner ES (2019). Parental control and Chinese adolescent smoking and drinking: The mediating role of refusal self-efficacy and the moderating role of sensation seeking. Child Youth Serv Rev..

[21] McCool JP, Cameron LD, Petrie KJ (2005). The influence of smoking imagery on the smoking intentions of young people: testing a media interpretation model. J Adolesc Heal..

[22] Dalton MA, Sargent JD, Beach ML, Titus-Ernstoff L, Gibson JJ, Ahrens MB (2003). Effect of viewing smoking in movies on adolescent smoking initiation: a cohort study. Lancet..

[23] Sargent JD, Beach ML, Adachi-Mejia AM, Gibson JJ, Titus-Ernstoff LT, Carusi CP (2005). Exposure to movie smoking: its relation to smoking initiation among US adolescents. Pediatrics..

[24] Bandura A (1991). Social cognitive theory of self-regulation. Organ Behav Hum Decis Process..

[25] Engels RCME, Hale WW, Noom M, De Vries H (2005). Self-efficacy and emotional adjustment as precursors of smoking in early adolescence. Subst Use Misuse..

[26] Hiemstra M, Otten R, de Leeuw RNH, van Schayck OCP, Engels RCME (2011). The changing role of self-efficacy in adolescent smoking initiation. J Adolesc Heal..

[27] Veselska Z, Madarasova Geckova A, Reijneveld SA, Van Dijk JP (2011). Self-efficacy, affectivity and smoking behavior in adolescence. Eur Addict Res..

[28] Ma H, Unger JB, Chou C-P, Sun P, Palmer PH, Zhou Y (2008). Risk factors for adolescent smoking in urban and rural China: findings from the China seven cities study. Addict Behav..

[29] Vitória PD, Salgueiro MF, Silva SA, de Vries H (2011). Social influence, intention to smoke, and adolescent smoking behaviour longitudinal relations. Br J Health Psychol..

[30] Smith BN, Bean MK, Mitchell KS, Speizer IS, Fries EA (2007). Psychosocial factors associated with non-smoking adolescents’ intentions to smoke. Health Educ Res..

[31] Halpern-Felsher BL, Biehl M, Kropp RY, Rubinstein ML (2004). Perceived risks and benefits of smoking: differences among adolescents with different smoking experiences and intentions. Prev Med.

[32] Aryal UR, Petzold M, Krettek A (2013). Perceived risks and benefits of cigarette smoking among Nepalese adolescents: a population-based cross-sectional study. BMC Public Health..

[33] Song AV, Morrell HER, Cornell JL, Ramos ME, Biehl M, Kropp RY (2009). Perceptions of smoking-related risks and benefits as predictors of adolescent smoking initiation. Am J Public Health..

[34] Harakeh Z, Scholte RHJ, de Vries H, Engels RCME (2006). Association between personality and adolescent smoking. Addict Behav..

[35] Goldberg LR (1990). An alternative “description of personality”: the big-five factor structure. J Pers Soc Psychol..

[36] de Leeuw RNH, Scholte RHJ, Sargent JD, Vermulst AA, Engels RCME (2010). Do interactions between personality and social-environmental factors explain smoking development in adolescence?. J Fam Psychol..

[37] Scal P, Ireland M, Borowsky IW (2003). Smoking among American adolescents: a risk and protective factor analysis. J Community Health..

[38] Piko BF, Luszczynska A, Gibbons FX, Tekozel M (2005). A culture-based study of personal and social influences of adolescent smoking. Eur J Public Health..

[39] Patton GC, Carlin JB, Coffey C, Wolfe R, Hibbert M, Bowes G (1998). Depression, anxiety, and smoking initiation: a prospective study over 3 years. Am J Public Health..

[40] Wilkinson D, Abraham C (2004). Constructing an integrated model of the antecedents of adolescent smoking. Br J Health Psychol..

[41] Giannakopoulos G, Tzavara C, Dimitrakaki C, Kolaitis G, Rotsika V, Tountas Y (2010). Emotional, behavioural problems and cigarette smoking in adolescence: findings of a Greek cross-sectional study. BMC Public Health..

[42] Topolski TD, Patrick DL, Edwards TC, Huebner CE, Connell FA, Mount KK (2001). Quality of life and health-risk behaviors among adolescents. J Adolesc Heal..

[43] Weiss JW, Palmer PH, Chou C-P, Mouttapa M, Johnson CA (2008). Association between psychological factors and adolescent smoking in seven cities in China. Int J Behav Med..

[44] Ajzen I (1991). The theory of planned behavior. Organ Behav Hum Decis Process..

[45] Hunter RF, Montes F, Murray JM, Sanchez-Franco SC, Montgomery SC, Jaramillo J, et al. MECHANISMS Study: using game theory to assess the effects of social norms and social networks on adolescent smoking in schools—study protocol. Front Public Heal. 2020;8. 10.3389/fpubh.2020.00377 PMC7417659 32850598

[46] Northern Ireland Statistics and Research Agency. Northern Ireland multiple deprivation measure 2017 (NIMDM2017) [Internet]. 2017 [cited 8 Oct 2020]. Available from: https://www.nisra.gov.uk/statistics/deprivation/northern-ireland-multiple-deprivation-measure-2017-nimdm2017.

[47] Colombian Institute for the Evaluation of Education (2017). Informe nacional de resultados del examen saber 11 - 2014-2 - 2016-2 [Internet].

[48] Cremers H-P, Mercken L, Oenema A, de Vries H (2012). A web-based computer-tailored smoking prevention programme for primary school children: intervention design and study protocol. BMC Public Health..

[49] Stigler MH, Perry CL, Arora M, Reddy KS (2006). Why are urban Indian 6th graders using more tobacco than 8th graders?. Findings from Project MYTRI. Tob Control..

[50] Dunne L, Thurston A, Gildea A, Kee F, Lazenbatt A (2016). Protocol: a randomised controlled trial evaluation of cancer focus NI’s ‘Dead Cool’ smoking prevention programme in post-primary schools. Int J Educ Res..

[51] Lawrance L (1989). Validation of a self-efficacy scale to predict adolescent smoking. Health Educ Res..

[52] Condiotte MM, Lichtenstein E (1981). Self-efficacy and relapse in smoking cessation programs. J Consult Clin Psychol..

[53] Ganley BJ, Rosario DI. The smoking attitudes, knowledge, intent, and behaviors of adolescents and young adults: implications for nursing practice. J Nurs Educ Pract. 2013;3(1).

[54] Leary MR, Kelly KM, Cottrell CA, Schreindorfer LS (2013). Construct validity of the need to belong scale: mapping the nomological network. J Pers Assess..

[55] Bevelander KE, Smit CR, van Woudenberg TJ, Buijs L, Burk WJ, Buijzen M (2018). Youth’s social network structures and peer influences: study protocol MyMovez project - Phase I. BMC Public Health..

[56] Collins KA, Westra HA, Dozois DJA, Stewart SH (2005). The validity of the brief version of the fear of negative evaluation scale. J Anxiety Disord..

[57] Leary MR (1983). A brief version of the fear of negative evaluation scale. Personal Soc Psychol Bull..

[58] Goodman R, Meltzer H, Bailey V (2003). The strengths and difficulties questionnaire: a pilot study on the validity of the self-report version. Int Rev Psychiatry..

[59] Morizot J (2014). Construct validity of adolescents’ self-reported big five personality traits: importance of conceptual breadth and initial validation of a short measure. Assessment..

[60] Ortet G, Martínez T, Mezquita L, Morizot J, Ibáñez MI (2017). Big five personality trait short questionnaire: preliminary validation with spanish adults. Span J Psychol..

[61] Rees G, Bradshaw J, Goswami H, Keung A (2010). Understanding children’s well-being: a national survey of young people’s well-being.

[62] Cohen J (1988). Statistical power analysis for the behavioral sciences.

[63] Chatterjee S, Price B (1991). Regression diagnostic.

[64] Action on Smoking and Health (2019). ASH fact sheet: tobacco and the developing world [Internet].

[65] Reidpath DD, Davey TM, Kadirvelu A, Soyiri IN, Allotey P (2014). Does one cigarette make an adolescent smoker, and is it influenced by age and age of smoking initiation? Evidence of association from the U.S. Youth Risk Behavior Surveillance System (2011). Prev Med.

[66] Su X, Li L, Griffiths SM, Gao Y, Lau JTF, Mo PKH (2015). Smoking behaviors and intentions among adolescents in rural China: the application of the theory of planned behavior and the role of social influence. Addict Behav..

[67] Xi B, Liang Y, Liu Y, Yan Y, Zhao M, Ma C (2016). Tobacco use and second-hand smoke exposure in young adolescents aged 12-15 years: data from 68 low-income and middle-income countries. Lancet Glob Heal..

[68] Ra JS, Cho YH (2017). Psychosocial factors associated with smoking intention in Korean male middle school students. J Sch Nurs..

[69] Villanti A, Boulay M, Juon H-S (2011). Peer, parent and media influences on adolescent smoking by developmental stage. Addict Behav..

[70] Morgenstern M, Sargent JD, Engels RCME, Florek E, Hanewinkel R (2013). Smoking in European adolescents: relation between media influences, family affluence, and migration background. Addict Behav..

[71] Mishu MP, Siddiqui F, Shukla R, Kanaan M, Dogar O, Siddiqi K (2021). Predictors of cigarette smoking, smokeless tobacco consumption, and use of both forms in adolescents in South Asia: a secondary analysis of the global youth tobacco surveys. Nicotine Tob Res..

[72] Weiss JW, Cen S, Schuster DV, Unger JB, Johnson CA, Mouttapa M (2006). Longitudinal effects of pro-tobacco and anti-tobacco messages on adolescent smoking susceptibility. Nicotine Tob Res..

[73] Cruz TB, McConnell R, Low BW, Unger JB, Pentz MA, Urman R (2019). Tobacco marketing and subsequent use of cigarettes, e-cigarettes, and hookah in adolescents. Nicotine Tob Res..

[74] Chang F-C, Lee C-M, Lai H-R, Chiang J-T, Lee P-H, Chen W-J (2006). Social influences and self-efficacy as predictors of youth smoking initiation and cessation: a 3-year longitudinal study of vocational high school students in Taiwan. Addiction..

[75] Mohammadi S, Ghajari H, Valizade R, Ghaderi N, Yousefi F, Taymoori P (2017). Predictors of smoking among the secondary high school boy students based on the health belief model. Int J Prev Med..

[76] Conner M, Grogan S, Fry G, Gough B, Higgins AR (2009). Direct, mediated and moderated impacts of personality variables on smoking initiation in adolescents. Psychol Health..

[77] Jung S, Choi E (2017). Life satisfaction and delinquent behaviors among Korean adolescents. Pers Individ Dif..

[78] Tomori M, Zalar B, Kores Plesnicar B, Ziherl S, Stergar E (2001). Smoking in relation to psychosocial risk factors in adolescents. Eur Child Adolesc Psychiatry..

[79] Lee LK, Paul CYC, Kam CW, Jagmohni K (2005). Smoking among secondary school students in Negeri Sembilan, Malaysia. Asia-Pacific J Public Heal.

[80] Dolcini MM, Adler NE, Ginsberg D (1996). Factors influencing agreement between self-reports and biological measures of smoking among adolescents. J Res Adolesc..

[81] Kandel DB, Schaffran C, Griesler PC, Hu M-C, Davies M, Benowitz N (2006). Salivary cotinine concentration versus self-reported cigarette smoking: three patterns of inconsistency in adolescence. Nicotine Tob Res..

[82] Yeager DS, Krosnick JA (2010). The validity of self-reported nicotine product use in the 2001-2008 National Health and Nutrition Examination Survey. Med Care..

[83] Miller DT, McFarland C (1991). When social comparison goes awry: The case of pluralistic ignorance. Social comparison: contemporary theory and research.

[84] McCormick LK, Crawford M, Anderson RH, Gittelsohn J, Kingsley B, Upson D (1999). Recruiting adolescents into qualitative tobacco research studies: experiences and lessons learned. J Sch Health..

[85] Swets JA (1988). Measuring the accuracy of diagnostic systems. Science..

[86] Spears R (2021). Social influence and group identity. Annu Rev Psychol..

