# Supplementary material for: Socio-environmental and psychosocial predictors of smoking susceptibility among adolescents with contrasting socio-cultural characteristics: a comparative analysis
Source: BMC Public Health. 2021 Dec 9;21:2240. doi: 10.1186/s12889-021-12351-x (PMC8662882; doi:10.1186/s12889-021-12351-x)

**Appendix**

**Survey Instruments**

***Socio-environmental Factors***

*Injunctive norms* were measured with seven items asking students to report if they believed important others would consider it appropriate for them to smoke and coded on a five-point scale from -2 = “definitely should smoke” to 2 = “definitely should not smoke”.

*Descriptive norms* were measured with five items that captured smoking of mother, father, brother, sister, and best friend on a five-point scale from 1 = “Very often” to 5 = “Never/Don’t know”. Three items were used to measure the proportion of friends, family, and classmates who smoke on a five-point scale from 1 = “Almost all of them” to 5 = “Almost none of them/Don’t know”.

*Exposure to advertising in media* was measured by asking if students had seen advertisements for tobacco in different media-related material and coded on a dichotomous scale (0 = “No”, 1 = “Yes”). Responses were summed to give an overall measure of exposure to advertising in media from 0 to 8.

*Exposure to tobacco advertising in shops* was measured using four items coded on a dichotomous scale asking students if they had seen advertisements for cigarettes in a supermarket, newsagent, petrol station, or other shops and summed to give an overall measure of exposure to advertising in shops from 0 to 4.

*School smoking information* was assessed with a single item asking, “Do you think your school has given you enough information on smoking?”, measured on a three point scale (1 = “No”, 2 = “Don’t know”, 3 = “Yes”).

***Smoking-related Cognitions***

*Self-efficacy* was assessed using the Lawrance (51) adaptation of the scales outlined in Condiotte & Lichtenstein (52) with three subscales: (i) emotional (9 items); (ii) friends (9 items); and (iii) opportunity (11 items). Students reported their capacity to resist smoking a cigarette on a six-point scale from 1 = “I am very sure I would smoke” to 6 = “I am very sure I would not smoke” in 29 different scenarios. A single overall score for self-efficacy was derived from these three subscales.

*Perceived behavioural control* (PBC) was assessed with two items: “Please indicate the extent to which you agree or disagree with the following statements…” (i) “If I smoked regularly, I'm sure that it would be easy for me to quit”; and (ii) “If I decided not to smoke, I am sure I could avoid smoking”. Responses were coded on a five-point Likert scale from 1 = “strongly disagree” to 5 = “strongly agree”.

*Perceived risks* of tobacco use were assessed with 13 items asking students to report the probability (between 0-100%) of 13 potential negative outcomes arising as a consequence of smoking 2-3 cigarettes a day. These outcomes were measured on three subscales corresponding to physical (7 items), social (3 items) and addiction (3 items) risks. An overall measure of perceived risk was derived from the three subscales.

*Perceived benefits* of tobacco use were assessed with 5 items asking students to report the probability (between 0-100%) of achieving five potential benefits of smoking 2-3 cigarettes a day. Benefits of smoking were measured on two subscales corresponding to physical (2 items) and social (3 items) benefits. An overall measure of perceived benefits was derived from the two subscales.

*Attitudes* towards smoking were assessed a 12-item scale outlined in Ganley and Rosario (53) asking students to rate their agreement with various statements related to smoking (e.g., “Smoking looks cool”) on a five-point Likert scale from 1 = “strongly disagree” to 5 = “strongly agree”. A higher score was indicative of more negative attitudes towards smoking. The scale was reversed for one item (“I do not like being around others who smoke”). An overall measure of attitudes towards smoking was derived by combining scores from each subscale.

*Knowledge* of health effects of smoking was assessed with six items outlined in Cremers et al (48) with students answering questions testing their knowledge of potential side effects associated with smoking. A score of 0 to 6 was derived as a sum of the number of correct answers provided by students.

***Psychosocial Characteristics and Personality Traits***

The *Need to Belong* score was derived from 10 items (e.g., “If other people don't seem to accept me, I don't let it bother me”) measured on a five-point scale, with a higher score indicating a greater need to belong.

The *Fear of Negative Evaluation* score was derived from 12 items (e.g., “I am afraid others will not approve of me”) measured on a five-point scale, with a higher score indicating a greater fear of negative evaluation.

The *Pro-Social Behaviour* score was derived from 5 items (e.g., “I try to be nice to other people. I care about their feelings”) measured on a three-point scale from 0 = “not true” to 2 = “certainly true”, with a higher score reflecting more prosocial behavioural preferences.

Self-perceived wellbeing was measured using five items (e.g., “My life is going well”) developed by the Children’s Society and based on Huebner’s life satisfaction scale. Responses were coded on a five-point Likert scale from 0 = “totally disagree” to 4 = “totally agree”, with a higher score indicating greater levels of self-perceived wellbeing. This scale was reversed for one item (“I wish I had a different kind of life”). An overall measure of wellbeing was derived from responses to the five items.

Truancy, and access to and disposal of pocket money were assessed using three separate items: (i) “have you ever stayed away from school without permission (truanted/bunked off)?”; (ii) “do you get regular pocket money (from parents, relatives)?”; and (iii) “can you spend your money however you like?”.

| Table 1: Description of multi-item scales to measure socio-environmental factors, smoking-related cognitions, and psychosocial traits | | | | | | | | | | | | | | | | | | |
| --- | --- | --- | --- | --- | --- | --- | --- | --- | --- | --- | --- | --- | --- | --- | --- | --- | --- | --- |
|  | Items | | Range | | Mean | | (SD) | | Example of item on scale | | | | | | | | | |
| **Socio-Environmental**  **Factors** | | | | | | | | | | | | | | | | | | |
| - **Injunctive Norms** |  | |  | |  | |  | |  | | | | | | | | | |
| - Important people | 1 | | -2-2 | | 1.75 | | (0.68) | | “Most of the people who are important to me think that I…Definitely should smoke” | | | | | | | | | |
| - Mother | 1 | | -2-2 | | 1.91 | | (0.38) | | “My mother thinks that I… Definitely should smoke” | | | | | | | | | |
| - Father | 1 | | -2-2 | | 1.73 | | (0.68) | | “My father thinks that I… Definitely should smoke” | | | | | | | | | |
| - Brother(s) | 1 | | -2-2 | | 1.40 | | (0.90) | | “My brother(s) think(s) that I… Definitely should smoke” | | | | | | | | | |
| - Sister(s) | 1 | | -2-2 | | 1.37 | | (0.93) | | “My sister(s) think(s) that I… Definitely should smoke” | | | | | | | | | |
| - Friends | 1 | | -2-2 | | 1.39 | | (0.93) | | “My friends think that I… Definitely should smoke” | | | | | | | | | |
| - Best friend | 1 | | -2-2 | | 1.56 | | (0.83) | | “My best friend thinks that I… Definitely should smoke” | | | | | | | | | |
| - **Descriptive Norms** |  | |  | |  | |  | |  | | | | | | | | | |
| - Best friend | 1 | | 1-5 | | 4.8 | | (0.72) | | Does your best friend smoke? | | | | | | | | | |
| - Mother | 1 | | 1-5 | | 4.44 | | (1.17) | | Does your mother smoke? | | | | | | | | | |
| - Father | 1 | | 1-5 | | 4.31 | | (1.30) | | Does your father smoke? | | | | | | | | | |
| - Brother(s) | 1 | | 1-5 | | 4.73 | | (0.85) | | Do any of your brothers smoke? | | | | | | | | | |
| - Sister(s) | 1 | | 1-5 | | 4.82 | | (0.71) | | Do any of your sisters smoke? | | | | | | | | | |
| - Friends | 1 | | 1-5 | | 4.72 | | (0.69) | | How many of your friends smoke? | | | | | | | | | |
| - Family | 1 | | 1-5 | | 4.31 | | (0.99) | | How many of your other family members smoke? | | | | | | | | | |
| - Classmates | 1 | | 1-5 | | 4.78 | | (0.55) | | How many of your classmates smoke? | | | | | | | | | |
| - School Smoking Information | 1 | | 1-3 | | 2.20 | | (0.88) | | “Do you think your school has given you enough information on smoking?” | | | | | | | | | |
| Exposure to Smoking in Media | 8 | | 0-8 | | 2.58 | | (2.03) | | “Have you seen any advertisements for tobacco… On television?” | | | | | | | | | |
| Exposure to Smoking in Shops | 4 | | 0-4 | | 2.19 | | (1.22) | | “In the past year, have you seen cigarette packets on display in any of the shops listed below?... A supermarket?” | | | | | | | | | |
| **Self-Efficacy** | | | | | | | | | | | | | | | | | | |
| - Emotion | 9 | | 1-6 | | 5.60 | | (0.81) | | “How sure are you that you could resist smoking cigarettes when… You are angry?” | | | | | | | | | |
| - Friends | 9 | | 1-6 | | 5.63 | | (0.78) | | “How sure are you that you could resist smoking cigarettes when… You are at a friend's house, no adults are home?” | | | | | | | | | |
| - Opportunity | 11 | | 1-6 | | 5.79 | | (0.59) | | “How sure are you that you could resist smoking cigarettes when… You are playing video games?” | | | | | | | | | |
| **Perceived Behavioural**  **Control** | | | | | | | | | | | | | | | | | | |
| Perceived Behavioural Control to Quit | 1 | | 1-5 | | 3.05 | | (1.44) | | “Please indicate the extent to which you agree or disagree with the following statements… If I smoked regularly, I'm sure that it would be easy for me to quit.” | | | | | | | | | |
| Perceived Behavioural Control to Avoid | 1 | | 1-5 | | 4.15 | | (1.20) | | “Please indicate the extent to which you agree or disagree with the following statements… If I decided not to smoke, I am sure I could avoid smoking.” | | | | | | | | | |
| **Perceived Risks of Smoking** |  | |  | |  | |  | |  | | | | | | | | | |
| - Physical | 7 | | 0-100 | | 60.50 | | (24.52) | | “Imagine that you just began smoking. You smoke about 2 or 3 cigarettes each day… Please estimate the chance you will get the following risks by using any percentage between 0% and 100%... You will get a bad cough from smoking?” | | | | | | | | | |
| - Social | 3 | | 0-100 | | 66.91 | | (27.28) | | “Imagine that you just began smoking. You smoke about 2 or 3 cigarettes each day… Please estimate the chance you will get the following risks by using any percentage between 0% and 100%... Your friends will be upset with you?” | | | | | | | | | |
| - Addiction | 3 | | 0-100 | | 38.82 | | (28.49) | | “Imagine that you just began smoking. You smoke about 2 or 3 cigarettes each day… Please estimate the chance you will get the following risks by using any percentage between 0% and 100%... You can quit smoking cigarettes if you want to?” | | | | | | | | | |
| **Perceived Benefits of Smoking** | |  | | | | | | | |  | | | | | | | | |
| - Physical | 2 | | 0-100 | | 32.30 | | (27.87) | | “Imagine that you just began smoking. You smoke about 2 or 3 cigarettes each day… Please estimate the chance you will get the following benefits by using any percentage between 0% and 100%... You will feel relaxed after smoking?” | | | | | | | | | |
| - Social | 2 | | 0-100 | | 19.32 | | (25.79) | | “Imagine that you just began smoking. You smoke about 2 or 3 cigarettes each day… Please estimate the chance you will get the following benefits by using any percentage between 0% and 100%... You will look cool?” | | | | | | | | | |
| **Attitudes Towards**  **Smoking** | | | | | | | | | | | | | | | | | | |
| Attitudes Towards Smoking | 12 | | 1-5 | | 3.93 | | (0.65) | | “Please indicate the extent to which you agree or disagree with the following statements… Smoking looks cool.” | | | | | | | | | |
| **Knowledge of Health**  **Effects** | | | | | | | | | | | | | | | | | | |
| Knowledge of Health Effects | 6 | | 0-6 | | 2.54 | | (1.48) | | “Please indicate whether you agree or disagree with the following statements… Smoking is only harmful when you smoke a lot.” | | | | | | | | | |
| **Pro-sociality** | | | | | | | | | | | | | | | | | | |
| - Need to Belong Scale | 9 | | 1-5 | | 2.86 | | (0.61) | | “Please indicate the degree to which each of the following statements is true or characteristic of you… I want other people to accept me.” | | | | | | | | | |
| - Fear of Negative Evaluation Scale | 11 | | 1-5 | | 2.76 | | (0.63) | | “Read each of the following statements carefully and indicate how characteristic it is of you according to the following scale… I worry about what other people will think of me even when I know it doesn't make any difference.” | | | | | | | | | |
| - Pro-social Behaviour Scale | 5 | | 0-10 | | 7.64 | | (2.15) | | “For each item, please mark the box for Not True, Somewhat True or Certainly True… I try to be nice to other people. I care about their feelings.” | | | | | | | | | |
| **Big 5 Personality**  **Traits** | | | | | | | | | | | | | | | | | | |
| - Openness | 10 | | 0-4 | | 2.56 | | (0.69) | | “Please say how much you agree or disagree with the following sentences. I see myself as someone who… Is original, often has new ideas.” | | | | | | | | | |
| - Extraversion | 10 | | 0-4 | | 2.63 | | (0.74) | | “Please say how much you agree or disagree with the following sentences. I see myself as someone who… Is a leader, capable of convincing others.” | | | | | | | | | |
| - Agreeableness | 10 | | 0-4 | | 2.58 | | (0.65) | | “Please say how much you agree or disagree with the following sentences. I see myself as someone who… Generally trusts others.” | | | | | | | | | |
| - Conscientiousness | 10 | | 0-4 | | 2.34 | | (0.66) | | “Please say how much you agree or disagree with the following sentences. I see myself as someone who… Does things efficiently, works well and quickly.” | | | | | | | | | |
| - Emotional Stability | 10 | | 0-4 | | 1.99 | | (0.76) | | “Please say how much you agree or disagree with the following sentences. I see myself as someone who… Is emotionally stable, not easily upset.” | | | | | | | | | |
| **Other Intra-personal**  **Factors** | | | | | | | | | | | | | | | | | | |
| - Wellbeing | 5 | | 0-20 | | 14.61 | | (4.71) | | “Please say how much you agree or disagree with the following sentences… My life is going well.” | | | | | | | | | |
| - Truancy | 1 | | 1-3 | | 2.54 | | (0.81) | | “Have you ever stayed away from school without permission (truanted/bunked off)?” | | | | | | | | | |
| - Pocket Money | 1 | | 1-2 | | 1.31 | | (0.46) | | “Do you get regular pocket money (from parents, relatives)?” | | | | | | | | | |
| - Pocket Money Spending | 1 | | 1-2 | | 1.34 | | (0.47) | | Can you spend your money however you like? | | | | | | | | | |
| Table 2: Unadjusted odds ratios | | | | | | | | | | | | | | | | |  |  |
|  | | | | Total | | | | | | | Northern Ireland | | | Bogotá | | |  |  |
| **Independent Variables** | | | | Crude OR | | 95% CI | | p-Value | | | Crude OR | 95% CI | p-Value | Crude OR | 95% CI | p-Value | [Variable] x Country p-value |  |
| **Socio-environmental Factors** | | | |  | | | | | | | | | | | | | |  |
| **Injunctive Norms** | | | |  |  |  |  |  |  |  |  |  |  |  |  |  |  |  |
| Important people | | | | 0.67 | | 0.60 - 0.75 | | 0.000 | | | 0.65 | 0.55 - 0.77 | 0.000 | 0.67 | 0.57 - 0.78 | 0.000 | 0.879 |  |
| Mother | | | | 0.79 | | 0.71 - 0.88 | | 0.000 | | | 0.78 | 0.67 - 0.91 | 0.002 | 0.81 | 0.70 - 0.93 | 0.004 | 0.494 |  |
| Father | | | | 0.85 | | 0.77 - 0.94 | | 0.001 | | | 0.85 | 0.73 - 0.99 | 0.039 | 0.88 | 0.77 - 1.01 | 0.071 | 0.518 |  |
| Brother(s) | | | | 0.83 | | 0.75 - 0.92 | | 0.000 | | | 0.72 | 0.61 - 0.84 | 0.000 | 0.92 | 0.80 - 1.05 | 0.216 | 0.024 |  |
| Sister(s) | | | | 0.83 | | 0.75 - 0.92 | | 0.000 | | | 0.77 | 0.65 - 0.90 | 0.001 | 0.88 | 0.77 - 1.00 | 0.058 | 0.185 |  |
| Friends | | | | 0.52 | | 0.47 - 0.58 | | 0.000 | | | 0.45 | 0.37 - 0.53 | 0.000 | 0.60 | 0.52 - 0.69 | 0.000 | 0.003 |  |
| Best friend | | | | 0.59 | | 0.53 - 0.66 | | 0.000 | | | 0.51 | 0.43 - 0.61 | 0.000 | 0.67 | 0.58 - 0.77 | 0.000 | 0.001 |  |
| **Descriptive Norms** | | | |  | | | | | | | | | | | | | |  |
| Best friend | | | | 0.63 | | 0.55 - 0.71 | | 0.000 | | | 0.49 | 0.40 - 0.62 | 0.000 | 0.73 | 0.63 - 0.85 | 0.000 | 0.013 |  |
| Mother | | | | 0.79 | | 0.71 - 0.87 | | 0.000 | | | 0.73 | 0.62 - 0.85 | 0.000 | 0.75 | 0.66 - 0.87 | 0.000 | 0.545 |  |
| Father | | | | 0.74 | | 0.67 - 0.82 | | 0.000 | | | 0.68 | 0.58 - 0.80 | 0.000 | 0.76 | 0.66 - 0.86 | 0.000 | 0.666 |  |
| Brother(s) | | | | 0.76 | | 0.68 - 0.84 | | 0.000 | | | 0.76 | 0.66 - 0.89 | 0.000 | 0.75 | 0.65 - 0.86 | 0.000 | 0.814 |  |
| Sister(s) | | | | 0.85 | | 0.77 - 0.94 | | 0.002 | | | 0.84 | 0.73 - 0.98 | 0.026 | 0.86 | 0.75 - 0.98 | 0.026 | 0.825 |  |
| Friends | | | | 0.55 | | 0.48 - 0.62 | | 0.000 | | | 0.46 | 0.37 - 0.57 | 0.000 | 0.60 | 0.50 - 0.71 | 0.000 | 0.073 |  |
| Family | | | | 0.67 | | 0.61 - 0.75 | | 0.000 | | | 0.59 | 0.50 - 0.69 | 0.000 | 0.69 | 0.60 - 0.79 | 0.000 | 0.285 |  |
| Classmates | | | | 0.76 | | 0.68 - 0.84 | | 0.000 | | | 0.64 | 0.54 - 0.76 | 0.000 | 0.82 | 0.72 - 0.95 | 0.006 | 0.186 |  |
| School Smoking Information | | | | 0.86 | | 0.77 - 0.95 | | 0.003 | | | 0.81 | 0.69 - 0.95 | 0.009 | 0.95 | 0.83 - 1.08 | 0.423 | 0.115 |  |
| Smoking in Media | | | | 1.39 | | 1.25 - 1.54 | | 0.000 | | | 1.52 | 1.29 - 1.78 | 0.000 | 1.24 | 1.08 - 1.42 | 0.002 | 0.137 |  |
| Smoking in Shops | | | | 1.19 | | 1.07 - 1.32 | | 0.001 | | | 1.20 | 1.01 - 1.42 | 0.033 | 1.28 | 1.11 - 1.47 | 0.000 | 0.426 |  |
| **Individual-Level Factors** | | | |  | | | | | | | | | | | | |  |  |
| **Smoking-Related Cognitions** | | | |  | |  | |  | | |  |  |  |  |  |  |  |  |
| Self-Efficacy | | | | 0.27 | | 0.23 - 0.33 | | 0.000 | | | 0.18 | 0.12 - 0.28 | 0.000 | 0.35 | 0.28 - 0.43 | 0.000 | 0.015 |  |
| Perceived Risks | | | | 0.63 | | 0.56 - 0.70 | | 0.000 | | | 0.71 | 0.60 - 0.83 | 0.000 | 0.64 | 0.55 - 0.73 | 0.000 | 0.520 |  |
| Perceived Benefits | | | | 1.26 | | 1.13 - 1.40 | | 0.000 | | | 1.59 | 1.34 - 1.88 | 0.000 | 1.08 | 0.94 - 1.23 | 0.275 | 0.000 |  |
| PBC Quit | | | | 1.04 | | 0.94 - 1.15 | | 0.493 | | | 0.99 | 0.84 - 1.17 | 0.944 | 0.92 | 0.80 - 1.05 | 0.197 | 0.441 |  |
| PBC Avoid | | | | 0.72 | | 0.65 - 0.80 | | 0.000 | | | 0.60 | 0.51 - 0.70 | 0.000 | 0.85 | 0.74 - 0.97 | 0.015 | 0.000 |  |
| Attitude | | | | 0.40 | | 0.35 - 0.46 | | 0.000 | | | 0.32 | 0.25 - 0.40 | 0.000 | 0.46 | 0.39 - 0.54 | 0.000 | 0.001 |  |
| Knowledge | | | | 0.80 | | 0.72 - 0.88 | | 0.000 | | | 1.06 | 0.90 - 1.24 | 0.506 | 0.72 | 0.62 - 0.82 | 0.000 | 0.000 |  |
| **Psychosocial Factors** | | | |  | |  | |  | | |  |  |  |  |  |  |  |  |
| Need to Belong | | | | 0.98 | | 0.88 - 1.08 | | 0.646 | | | 0.98 | 0.83 - 1.15 | 0.773 | 1.10 | 0.96 - 1.26 | 0.167 | 0.274 |  |
| Fear of Negative Evaluation | | | | 1.11 | | 1.00 - 1.23 | | 0.058 | | | 1.19 | 1.00 - 1.40 | 0.044 | 1.21 | 1.05 - 1.38 | 0.007 | 0.568 |  |
| Prosociality | | | | 0.73 | | 0.66 - 0.81 | | 0.000 | | | 0.61 | 0.52 - 0.72 | 0.000 | 0.88 | 0.77 - 1.00 | 0.054 | 0.001 |  |
| Openness | | | | 0.72 | | 0.64 - 0.81 | | 0.000 | | | 0.64 | 0.54 - 0.76 | 0.000 | 0.72 | 0.61 - 0.85 | 0.000 | 0.233 |  |
| Extraversion | | | | 0.87 | | 0.78 - 0.98 | | 0.021 | | | 0.91 | 0.77 - 1.08 | 0.287 | 0.80 | 0.68 - 0.94 | 0.006 | 0.190 |  |
| Agreeableness | | | | 0.60 | | 0.53 - 0.68 | | 0.000 | | | 0.49 | 0.40 - 0.60 | 0.000 | 0.67 | 0.57 - 0.79 | 0.000 | 0.020 |  |
| Conscientiousness | | | | 0.58 | | 0.51 - 0.66 | | 0.000 | | | 0.47 | 0.38 - 0.59 | 0.000 | 0.64 | 0.54 - 0.76 | 0.000 | 0.028 |  |
| Stability | | | | 0.75 | | 0.66 - 0.84 | | 0.000 | | | 0.68 | 0.56 - 0.82 | 0.000 | 0.74 | 0.63 - 0.88 | 0.000 | 0.725 |  |
| Wellbeing | | | | 0.59 | | 0.53 - 0.66 | | 0.000 | | | 0.56 | 0.47 - 0.66 | 0.000 | 0.63 | 0.55 - 0.73 | 0.000 | 0.193 |  |
| Truancy | | | | 0.58 | | 0.53 - 0.65 | | 0.000 | | | 0.61 | 0.52 - 0.71 | 0.000 | 0.60 | 0.52 - 0.69 | 0.000 | 0.104 |  |
| Pocket Money | | | | 0.95 | | 0.86 - 1.06 | | 0.372 | | | 1.03 | 0.88 - 1.22 | 0.712 | 0.94 | 0.82 - 1.07 | 0.340 | 0.364 |  |
| Pocket Money Spending | | | | 0.85 | | 0.76 - 0.94 | | 0.002 | | | 0.90 | 0.76 - 1.06 | 0.197 | 0.82 | 0.71 - 0.94 | 0.004 | 0.402 |  |
| **Sociodemographic Factors** | | | |  | | | | | | | | | | | | | |  |
| Country | | | | 1.67 | | 1.36 - 2.06 | | 0.000 | | |  |  |  |  |  |  |  |  |
| Gender | | | | 0.97 | | 0.80 - 1.18 | | 0.781 | | | 0.97 | 0.72 - 1.32 | 0.857 | 1.00 | 0.77 - 1.30 | 0.986 | 0.470 |  |
| Age | | | | 1.46 | | 1.29 - 1.65 | | 0.000 | | | 1.28 | 0.93 - 1.75 | 0.132 | 1.41 | 1.23 - 1.62 | 0.000 | 0.565 |  |
| Socioeconomic Status | | | |  | |  | |  | | | 0.91 | 0.77 - 1.09 | 0.306 | 0.86 | 0.75 - 0.99 | 0.035 |  |  |
| School Socioeconomic Status | | | |  | |  | |  | | | 0.89 | 0.75 - 1.04 | 0.148 | 0.83 | 0.73 - 0.95 | 0.008 |  |  |
| Single Parent | | | | 1.23 | | 0.99 - 1.54 | | 0.065 | | | 1.20 | 0.80 - 1.80 | 0.387 | 1.05 | 0.80 - 1.38 | 0.738 | 0.580 |  |

| Table 3: Adjusted odds ratios (adjusted for sociodemographic factors) | | | | | | | | | | |
| --- | --- | --- | --- | --- | --- | --- | --- | --- | --- | --- |
|  | Total^1^ | | | Northern Ireland^2^ | | | Bogotá^2^ | | |  |
| **Independent Variables** | OR | 95% CI | p-Value | OR | 95% CI | p-Value | OR | 95% CI | p-Value | [Variable] x Country p-value |
| **Socio-environmental Factors** |  | | | | | | | | | |
| **Injunctive Norms** |  |  |  |  |  |  |  |  |  |  |
| Important people | 0.67 | 0.60 - 0.75 | 0.000 | 0.64 | 0.54 - 0.77 | 0.000 | 0.68 | 0.58 - 0.80 | 0.000 | 0.894 |
| Mother | 0.80 | 0.72 - 0.90 | 0.000 | 0.81 | 0.68 - 0.97 | 0.020 | 0.83 | 0.72 - 0.97 | 0.019 | 0.476 |
| Father | 0.88 | 0.80 - 0.98 | 0.023 | 0.85 | 0.70 - 1.02 | 0.079 | 0.90 | 0.78 - 1.04 | 0.162 | 0.521 |
| Brother(s) | 0.82 | 0.74 - 0.91 | 0.000 | 0.67 | 0.56 - 0.79 | 0.000 | 0.91 | 0.79 - 1.05 | 0.183 | 0.027 |
| Sister(s) | 0.83 | 0.75 - 0.92 | 0.000 | 0.76 | 0.65 - 0.91 | 0.002 | 0.85 | 0.74 - 0.97 | 0.019 | 0.235 |
| Friends | 0.54 | 0.48 - 0.60 | 0.000 | 0.43 | 0.36 - 0.52 | 0.000 | 0.61 | 0.53 - 0.71 | 0.000 | 0.003 |
| Best friend | 0.61 | 0.54 - 0.68 | 0.000 | 0.49 | 0.41 - 0.59 | 0.000 | 0.67 | 0.58 - 0.77 | 0.000 | 0.001 |
| **Descriptive Norms** |  | | | | | | | | | |
| Best friend | 0.63 | 0.55 - 0.71 | 0.000 | 0.48 | 0.38 - 0.62 | 0.000 | 0.77 | 0.66 - 0.90 | 0.001 | 0.009 |
| Mother | 0.74 | 0.67 - 0.83 | 0.000 | 0.73 | 0.62 - 0.86 | 0.000 | 0.76 | 0.66 - 0.88 | 0.000 | 0.658 |
| Father | 0.72 | 0.65 - 0.80 | 0.000 | 0.67 | 0.57 - 0.80 | 0.000 | 0.77 | 0.67 - 0.88 | 0.000 | 0.498 |
| Brother(s) | 0.76 | 0.68 - 0.84 | 0.000 | 0.73 | 0.62 - 0.86 | 0.000 | 0.77 | 0.67 - 0.90 | 0.001 | 0.995 |
| Sister(s) | 0.85 | 0.77 - 0.95 | 0.003 | 0.82 | 0.70 - 0.97 | 0.018 | 0.88 | 0.76 - 1.01 | 0.070 | 0.647 |
| Friends | 0.55 | 0.48 - 0.62 | 0.000 | 0.47 | 0.38 - 0.59 | 0.000 | 0.63 | 0.53 - 0.76 | 0.000 | 0.035 |
| Family | 0.65 | 0.58 - 0.73 | 0.000 | 0.59 | 0.49 - 0.70 | 0.000 | 0.71 | 0.61 - 0.83 | 0.000 | 0.168 |
| Classmates | 0.73 | 0.65 - 0.81 | 0.000 | 0.62 | 0.51 - 0.74 | 0.000 | 0.84 | 0.73 - 0.97 | 0.018 | 0.141 |
| School Smoking Information | 0.90 | 0.81 - 1.00 | 0.045 | 0.79 | 0.66 - 0.93 | 0.006 | 0.98 | 0.85 - 1.12 | 0.750 | 0.078 |
| Smoking in Media | 1.34 | 1.21 - 1.49 | 0.000 | 1.62 | 1.36 - 1.94 | 0.000 | 1.22 | 1.06 - 1.40 | 0.006 | 0.112 |
| Smoking in Shops | 1.23 | 1.10 - 1.37 | 0.000 | 1.22 | 1.02 - 1.47 | 0.028 | 1.26 | 1.09 - 1.46 | 0.001 | 0.641 |
| **Individual-Level Factors** |  | | | | | | | | |  |
| **Smoking-Related Cognitions** |  | | | | | | | | |  |
| Self-Efficacy | 0.29 | 0.24 - 0.35 | 0.000 | 0.19 | 0.12 - 0.28 | 0.000 | 0.37 | 0.30 - 0.46 | 0.000 | 0.010 |
| Perceived Risks | 0.66 | 0.59 - 0.74 | 0.000 | 0.67 | 0.56 - 0.81 | 0.000 | 0.66 | 0.57 - 0.76 | 0.000 | 0.591 |
| Perceived Benefits | 1.27 | 1.14 - 1.41 | 0.000 | 1.60 | 1.33 - 1.91 | 0.000 | 1.11 | 0.97 - 1.28 | 0.139 | 0.000 |
| PBC Quit | 0.94 | 0.84 - 1.05 | 0.268 | 0.98 | 0.82 - 1.16 | 0.783 | 0.89 | 0.78 - 1.03 | 0.116 | 0.392 |
| PBC Avoid | 0.74 | 0.66 - 0.82 | 0.000 | 0.54 | 0.45 - 0.65 | 0.000 | 0.85 | 0.74 - 0.98 | 0.022 | 0.000 |
| Attitude | 0.41 | 0.36 - 0.47 | 0.000 | 0.26 | 0.20 - 0.34 | 0.000 | 0.49 | 0.42 - 0.58 | 0.000 | 0.001 |
| Knowledge | 0.85 | 0.76 - 0.95 | 0.003 | 1.03 | 0.87 - 1.23 | 0.732 | 0.73 | 0.63 - 0.84 | 0.000 | 0.000 |
| **Psychosocial Factors** |  | | | | | | | | | |
| Need to Belong | 1.07 | 0.96 - 1.19 | 0.263 | 1.00 | 0.84 - 1.19 | 0.988 | 1.11 | 0.96 - 1.27 | 0.157 | 0.215 |
| Fear of Negative Evaluation | 1.22 | 1.09 - 1.36 | 0.001 | 1.15 | 0.96 - 1.38 | 0.124 | 1.20 | 1.04 - 1.38 | 0.011 | 0.500 |
| Prosociality | 0.74 | 0.67 - 0.83 | 0.000 | 0.55 | 0.46 - 0.67 | 0.000 | 0.87 | 0.76 - 1.00 | 0.050 | 0.000 |
| Openness | 0.67 | 0.59 - 0.76 | 0.000 | 0.58 | 0.47 - 0.71 | 0.000 | 0.71 | 0.60 - 0.84 | 0.000 | 0.237 |
| Extraversion | 0.84 | 0.75 - 0.95 | 0.005 | 0.89 | 0.74 - 1.07 | 0.208 | 0.79 | 0.67 - 0.94 | 0.006 | 0.175 |
| Agreeableness | 0.60 | 0.52 - 0.68 | 0.000 | 0.50 | 0.40 - 0.62 | 0.000 | 0.69 | 0.58 - 0.82 | 0.000 | 0.007 |
| Conscientiousness | 0.57 | 0.50 - 0.66 | 0.000 | 0.46 | 0.37 - 0.58 | 0.000 | 0.65 | 0.54 - 0.77 | 0.000 | 0.015 |
| Stability | 0.70 | 0.62 - 0.80 | 0.000 | 0.69 | 0.56 - 0.84 | 0.000 | 0.75 | 0.63 - 0.90 | 0.001 | 0.610 |
| Wellbeing | 0.61 | 0.54 - 0.68 | 0.000 | 0.54 | 0.45 - 0.65 | 0.000 | 0.65 | 0.56 - 0.75 | 0.000 | 0.114 |
| Truancy | 0.62 | 0.55 - 0.69 | 0.000 | 0.61 | 0.51 - 0.72 | 0.000 | 0.64 | 0.55 - 0.74 | 0.000 | 0.057 |
| Pocket Money | 0.97 | 0.87 - 1.08 | 0.603 | 1.02 | 0.86 - 1.22 | 0.797 | 0.93 | 0.81 - 1.07 | 0.328 | 0.310 |
| Pocket Money Spending | 0.86 | 0.77 - 0.96 | 0.006 | 0.88 | 0.73 - 1.05 | 0.151 | 0.84 | 0.73 - 0.97 | 0.016 | 0.484 |
| ^1^ Adjusted for Country, Gender, Age, Single Parent  ^2^ Adjusted for Gender, Age, Single Parent, Socioeconomic Status and School Socioeconomic Status | | | | | | | | | | |

| Table 4: Multivariate-adjusted odds ratios | | | | | | | | | |  |
| --- | --- | --- | --- | --- | --- | --- | --- | --- | --- | --- |
|  | Total^1^ | | | Northern Ireland^2^ | | | Bogotá^2^ | | |  |
| **Independent Variables** | Adjusted OR | 95% CI | p-Value | Adjusted OR | 95% CI | p-Value | Adjusted OR | 95% CI | p-Value | [Variable] x Country p-value |
| **Socio-environmental Factors** |  | | | | | | | | | |
| **Injunctive Norms** |  |  |  |  |  |  |  |  |  |  |
| Important people | 0.86 | 0.76 - 0.97 | 0.014 | 0.88 | 0.66 - 1.18 | 0.395 | 0.82 | 0.66 - 1.02 | 0.078 | 0.346 |
| Mother | 1.06 | 0.97 - 1.16 | 0.194 | 1.41 | 0.99 - 2.01 | 0.056 | 1.04 | 0.85 - 1.27 | 0.712 | 0.540 |
| Father | 1.15 | 1.00 - 1.33 | 0.044 | 1.21 | 0.87 - 1.68 | 0.262 | 1.08 | 0.94 - 1.25 | 0.295 | 0.205 |
| Brother(s) | 0.99 | 0.86 - 1.13 | 0.856 | 0.90 | 0.67 - 1.22 | 0.513 | 1.10 | 0.84 - 1.46 | 0.478 | 0.545 |
| Sister(s) | 0.94 | 0.91 - 0.98 | 0.002 | 0.87 | 0.64 - 1.19 | 0.387 | 0.93 | 0.74 - 1.17 | 0.530 | 0.979 |
| Friends | 0.79 | 0.76 - 0.82 | 0.000 | 0.74 | 0.27 - 1.99 | 0.548 | 0.81 | 0.61 - 1.07 | 0.136 | 0.761 |
| Best friend | 1.04 | 0.99 - 1.10 | 0.134 | 1.23 | 0.40 - 3.72 | 0.718 | 1.00 | 0.80 - 1.25 | 0.975 | 0.738 |
| **Descriptive Norms** |  | | | | | | | | | |
| Best friend | 0.94 | 0.75 - 1.17 | 0.560 | 0.71 | 0.21 - 2.32 | 0.566 | 1.00 | 0.81 - 1.23 | 0.985 | 0.212 |
| Mother | 1.03 | 0.88 - 1.19 | 0.746 | 1.37 | 1.06 - 1.76 | 0.016 | 0.92 | 0.78 - 1.09 | 0.346 | 0.385 |
| Father | 0.88 | 0.76 - 1.03 | 0.105 | 0.87 | 0.66 - 1.15 | 0.320 | 0.81 | 0.62 - 1.06 | 0.125 | 0.529 |
| Brother(s) | 0.83 | 0.68 - 1.01 | 0.063 | 0.92 | 0.65 - 1.30 | 0.627 | 0.75 | 0.52 - 1.08 | 0.121 | 0.172 |
| Sister(s) | 1.12 | 1.06 - 1.18 | 0.000 | 0.99 | 0.41 - 2.37 | 0.979 | 1.08 | 0.96 - 1.22 | 0.208 | 0.480 |
| Friends | 0.90 | 0.80 - 1.02 | 0.097 | 0.95 | 0.45 - 2.01 | 0.889 | 0.86 | 0.76 - 0.98 | 0.028 | 0.338 |
| Family | 0.82 | 0.62 - 1.07 | 0.139 | 0.64 | 0.41 - 1.00 | 0.049 | 0.94 | 0.70 - 1.26 | 0.686 | 0.292 |
| Classmates | 0.96 | 0.91 - 1.02 | 0.180 | 0.89 | 0.60 - 1.33 | 0.568 | 0.98 | 0.78 - 1.22 | 0.830 | 0.395 |
| School Smoking Information | 0.96 | 0.76 - 1.20 | 0.702 | 0.75 | 0.59 - 0.96 | 0.024 | 1.09 | 0.92 - 1.29 | 0.313 | 0.039 |
| Smoking in Media | 1.04 | 0.99 - 1.09 | 0.085 | 1.10 | 0.91 - 1.34 | 0.326 | 1.05 | 0.80 - 1.38 | 0.720 | 0.843 |
| Smoking in Shops | 1.07 | 1.03 - 1.11 | 0.000 | 0.98 | 0.64 - 1.50 | 0.910 | 1.10 | 0.96 - 1.25 | 0.173 | 0.913 |
| **Individual-Level Factors** |  | | | | | | | | |  |
| **Smoking-Related Cognitions** |  | | | | | | | | |  |
| Self-Efficacy | 0.59 | 0.53 - 0.65 | 0.000 | 0.62 | 0.37 - 1.03 | 0.067 | 0.58 | 0.40 - 0.83 | 0.003 | 0.996 |
| Perceived Risks | 0.86 | 0.85 - 0.86 | 0.000 | 0.92 | 0.66 - 1.30 | 0.646 | 0.85 | 0.67 - 1.08 | 0.187 | 0.822 |
| Perceived Benefits | 1.02 | 0.95 - 1.10 | 0.582 | 1.02 | 0.75 - 1.40 | 0.885 | 1.02 | 0.85 - 1.22 | 0.845 | 0.177 |
| PBC Quit | 0.83 | 0.64 - 1.08 | 0.168 | 0.94 | 0.58 - 1.52 | 0.792 | 0.71 | 0.56 - 0.90 | 0.005 | 0.206 |
| PBC Avoid | 0.96 | 0.91 - 1.01 | 0.118 | 0.91 | 0.56 - 1.50 | 0.722 | 1.00 | 0.78 - 1.28 | 0.979 | 0.911 |
| Attitude | 0.62 | 0.47 - 0.80 | 0.000 | 0.35 | 0.23 - 0.51 | 0.000 | 0.68 | 0.43 - 1.08 | 0.100 | 0.030 |
| Knowledge | 0.95 | 0.73 - 1.24 | 0.712 | 0.99 | 0.68 - 1.45 | 0.969 | 0.84 | 0.66 - 1.07 | 0.159 | 0.215 |
| **Psychosocial Factors** |  | | | | | | | | | |
| Need to Belong | 1.09 | 1.02 - 1.16 | 0.010 | 1.15 | 0.85 - 1.56 | 0.369 | 1.09 | 0.85 - 1.40 | 0.497 | 0.541 |
| Fear of Negative Evaluation | 1.10 | 0.96 - 1.27 | 0.184 | 1.02 | 0.59 - 1.77 | 0.943 | 1.03 | 0.79 - 1.36 | 0.810 | 0.942 |
| Prosociality | 0.95 | 0.95 - 0.96 | 0.000 | 1.01 | 0.67 - 1.51 | 0.964 | 1.00 | 0.80 - 1.26 | 0.979 | 0.051 |
| Openness | 0.79 | 0.56 - 1.12 | 0.191 | 0.59 | 0.50 - 0.69 | 0.000 | 0.91 | 0.61 - 1.35 | 0.636 | 0.053 |
| Extraversion | 1.07 | 0.70 - 1.64 | 0.756 | 1.40 | 1.04 - 1.90 | 0.027 | 0.88 | 0.77 - 1.01 | 0.076 | 0.530 |
| Agreeableness | 0.92 | 0.64 - 1.34 | 0.670 | 0.69 | 0.39 - 1.22 | 0.207 | 1.10 | 0.71 - 1.70 | 0.676 | 0.002 |
| Conscientiousness | 0.92 | 0.90 - 0.94 | 0.000 | 1.01 | 0.64 - 1.58 | 0.972 | 0.95 | 0.73 - 1.22 | 0.672 | 0.056 |
| Stability | 0.97 | 0.92 - 1.02 | 0.209 | 1.01 | 0.75 - 1.36 | 0.954 | 0.94 | 0.83 - 1.07 | 0.367 | 0.497 |
| Wellbeing | 0.89 | 0.66 - 1.18 | 0.404 | 0.57 | 0.44 - 0.74 | 0.000 | 0.95 | 0.72 - 1.26 | 0.726 | 0.031 |
| Truancy | 0.72 | 0.67 - 0.78 | 0.000 | 0.91 | 0.60 - 1.39 | 0.662 | 0.69 | 0.52 - 0.92 | 0.010 | 0.900 |
| Pocket Money | 1.01 | 0.76 - 1.34 | 0.934 | 1.20 | 1.06 - 1.37 | 0.004 | 0.87 | 0.70 - 1.07 | 0.183 | 0.030 |
| Pocket Money Spending | 0.92 | 0.89 - 0.94 | 0.000 | 0.89 | 0.61 - 1.29 | 0.535 | 0.95 | 0.83 - 1.10 | 0.532 | 0.989 |
| **Sociodemographic Factors** |  | | | | | | | | | |
| Country | 1.50 | 1.04 - 2.15 | 0.030 |  |  |  |  |  |  |  |
| Gender | 0.92 | 0.81 - 1.05 | 0.231 | 0.89 | 0.35 - 2.26 | 0.808 | 1.01 | 0.64 - 1.60 | 0.969 | 0.348 |
| Age | 1.04 | 1.03 - 1.04 | 0.000 | 1.18 | 0.66 - 2.11 | 0.585 | 1.10 | 0.95 - 1.27 | 0.190 | 0.645 |
| Socioeconomic Status |  |  |  | 0.98 | 0.57 - 1.68 | 0.935 | 1.08 | 0.86 - 1.38 | 0.503 |  |
| School Socioeconomic Status |  |  |  | 1.20 | 0.79 - 1.82 | 0.397 | 0.95 | 0.75 - 1.21 | 0.690 |  |
| Single Parent | 1.02 | 0.49 - 2.12 | 0.964 | 0.46 | 0.15 - 1.39 | 0.170 | 1.31 | 0.71 - 2.40 | 0.384 | 0.010 |
| ^1^ Adjusted for clustering at country level  ^2^ Adjusted for clustering at school level | | | | | | | | | | |

Table 5: Pearson’s product-moment correlation matrix of independent variables (excluding sociodemographic factors)

|  | Injunctive~1 | Injunctive~2 | Injunctive~3 | Injunctive~4 | Injunctive~5 | Injunctive~6 | Injunctive~7 |
| --- | --- | --- | --- | --- | --- | --- | --- |
| Inj_Important people1 | 1.0000 |  |  |  |  |  |  |
| Inj_Mother2 | 0.3642* | 1.0000 |  |  |  |  |  |
| Inj_Father3 | 0.2234* | 0.3931* | 1.0000 |  |  |  |  |
| Inj_Brother(s)4 | 0.2092* | 0.2660* | 0.2762* | 1.0000 |  |  |  |
| Inj_Sister(s)5 | 0.1784* | 0.2591* | 0.2702* | 0.3601* | 1.0000 |  |  |
| Inj_Friends6 | 0.3473* | 0.2539* | 0.2220* | 0.3067* | 0.2435* | 1.0000 |  |
| Inj_Best friend7 | 0.3572* | 0.2832* | 0.2283* | 0.2751* | 0.2387* | 0.6195* | 1.0000 |
| Desc_Best friend1 | 0.0997* | 0.1180* | 0.0879* | 0.0117 | 0.0252 | 0.2166* | 0.1923* |
| Desc_Mother 2 | 0.0794* | 0.0618* | 0.0246 | 0.0575* | 0.0054 | 0.0836* | 0.0573* |
| Desc_Father3 | 0.0876* | 0.0155 | -0.0054 | 0.0791* | 0.0169 | 0.1097* | 0.0903* |
| Desc_Brother(s)4 | 0.0158 | 0.0112 | 0.0324 | -0.0482 | -0.0036 | 0.0343 | 0.0202 |
| Desc_Sister(s)5 | 0.0397 | 0.0112 | 0.0230 | 0.0121 | -0.0392 | 0.0718* | 0.0518* |
| Desc_Friends6 | 0.1111* | 0.1128* | 0.0737* | 0.0488 | 0.0439 | 0.2713* | 0.2008* |
| Desc_Family7 | 0.0518* | 0.0815* | 0.0819* | 0.0642* | 0.0286 | 0.1370* | 0.1055* |
| Desc_Classmates8 | 0.0421 | 0.0356 | 0.0096 | 0.0479 | 0.0234 | 0.1339* | 0.0614* |
| School Info | 0.0590* | 0.0253 | 0.0805* | 0.0218 | 0.0116 | 0.0682* | 0.0963* |
| Advertising Media | -0.0804* | -0.0780* | -0.0786* | -0.0420 | 0.0084 | -0.1426* | -0.1311* |
| Advertising Shops | -0.0204 | 0.0291 | -0.0159 | -0.0735* | 0.0032 | -0.0708* | -0.0675* |
| Self Efficacy | 0.2066* | 0.1394* | 0.1710* | 0.0812* | 0.0999* | 0.2921* | 0.2728* |
| Risk | 0.1066* | 0.0869* | 0.1029* | 0.0494 | 0.0805* | 0.1842* | 0.1870* |
| Benefits | -0.0269 | -0.0089 | 0.0125 | -0.0539* | -0.0236 | -0.1087* | -0.1088* |
| PBC Quit | 0.0065 | -0.0274 | -0.0568* | 0.0085 | 0.0171 | -0.0107 | -0.0398 |
| PBC Avoid | 0.1199* | 0.0968* | 0.1023* | 0.0207 | 0.0392 | 0.1110* | 0.0992* |
| Attitude | 0.2102* | 0.1756* | 0.1405* | 0.1054* | 0.1062* | 0.2924* | 0.2657* |
| Knowledge | 0.0772* | 0.0883* | 0.0958* | 0.0200 | 0.0433 | 0.0882* | 0.0983* |
| Belong | -0.0341 | -0.0092 | 0.0175 | 0.0080 | -0.0027 | 0.0347 | 0.0567* |
| Negative | -0.0542* | 0.0292 | -0.0306 | 0.0452 | 0.0180 | -0.0175 | 0.0072 |
| Prosocial | 0.1160* | 0.0951* | 0.0818* | 0.0805* | 0.0400 | 0.1766* | 0.1947* |
| Big5Open | 0.0848* | 0.0548 | 0.0364 | 0.0484 | 0.0156 | 0.0448 | 0.0422 |
| Big5Extra | 0.0834* | 0.0204 | 0.0521 | 0.0298 | 0.0033 | 0.0514 | 0.0331 |
| Big5Agree | 0.1432* | 0.0900* | 0.0598* | 0.0574* | 0.0292 | 0.1986* | 0.1492* |
| Big5Cons | 0.1174* | 0.0433 | 0.0484 | 0.0471 | 0.0160 | 0.1946* | 0.1601* |
| Big5Stab | 0.0850* | 0.0034 | 0.0346 | 0.0191 | 0.0153 | 0.0916* | 0.0442 |
| Wellbeing | 0.1548* | 0.0777* | 0.1707* | 0.0946* | 0.0821* | 0.2193* | 0.2243* |
| Truancy | 0.0974* | 0.0755* | 0.1278* | 0.0310 | 0.0647* | 0.2137* | 0.2079* |
| Pocket Money | -0.0314 | 0.0044 | -0.0091 | 0.0163 | 0.0030 | 0.0048 | -0.0202 |
| Pocket Money Spend | -0.0381 | -0.0257 | -0.0137 | 0.0083 | 0.0098 | 0.0604* | 0.0517* |
| Country | 0.0182 | -0.0365 | -0.1195* | 0.0174 | -0.0185 | -0.0883* | -0.1338* |
| Gender | -0.0191 | 0.0234 | -0.0594* | 0.0110 | -0.0285 | 0.0302 | 0.0628* |
| Age | -0.0639* | -0.0705* | -0.0907* | 0.0080 | -0.0005 | -0.1186* | -0.0867* |
| Single Parent | -0.0352 | -0.0664* | -0.2740* | -0.0222 | -0.0401 | -0.0476 | -0.0531* |

|  | Descriptive~1 | Descriptive~2 | Descriptive~3 | Descriptive~4 | Descriptive~5 | Descriptive~6 | Descriptive~7 |
| --- | --- | --- | --- | --- | --- | --- | --- |
| Desc_Best friend1 | 1.0000 |  |  |  |  |  |  |
| Desc_Mother 2 | 0.1985* | 1.0000 |  |  |  |  |  |
| Desc_Father3 | 0.1081* | 0.3622* | 1.0000 |  |  |  |  |
| Desc_Brother(s)4 | 0.1845* | 0.1848* | 0.1462* | 1.0000 |  |  |  |
| Desc_Sister(s)5 | 0.1968* | 0.2011* | 0.1170* | 0.2137* | 1.0000 |  |  |
| Desc_Friends6 | 0.4575* | 0.1275* | 0.1571* | 0.1507* | 0.1308* | 1.0000 |  |
| Desc_Family7 | 0.2023* | 0.4073* | 0.3526* | 0.2028* | 0.1628* | 0.2584* | 1.0000 |
| Desc_Classmates8 | 0.2691* | 0.0770* | 0.0864* | 0.0505* | 0.0996* | 0.3958* | 0.1635* |
| School Info | 0.0642* | -0.0206 | -0.0081 | 0.0232 | 0.0160 | 0.0391 | -0.0145 |
| Advertising Media | -0.1695* | -0.0728* | -0.0681* | -0.0878* | -0.1544* | -0.1613* | -0.1448* |
| Advertising Shops | -0.0790* | -0.1018* | -0.0956* | -0.0795* | -0.0444 | -0.0984* | -0.1251* |
| Self Efficacy | 0.3350* | 0.1632* | 0.1540* | 0.2002* | 0.1599* | 0.3472* | 0.1907* |
| Risk | 0.0954* | -0.0211 | 0.0386 | 0.0421 | 0.0355 | 0.1270* | 0.0132 |
| Benefits | -0.0923* | -0.0161 | -0.0056 | -0.0489 | -0.0435 | -0.1113* | -0.0746* |
| PBC Quit | -0.0229 | 0.0281 | 0.0255 | -0.0279 | -0.0246 | -0.0190 | 0.0447 |
| PBC Avoid | 0.0630* | -0.0227 | -0.0040 | 0.0148 | 0.0123 | 0.0889* | 0.0027 |
| Attitude | 0.2038* | 0.1105* | 0.1140* | 0.0774* | 0.0516* | 0.2738* | 0.1546* |
| Knowledge | 0.0288 | -0.0410 | -0.0269 | -0.0191 | 0.0184 | -0.0058 | -0.0741* |
| Belong | -0.0226 | -0.0233 | -0.0345 | 0.0295 | 0.0013 | 0.0164 | -0.0194 |
| Negative | -0.0438 | -0.0754* | -0.0358 | -0.0107 | -0.0311 | -0.0229 | -0.0562* |
| Prosocial | 0.0914* | 0.0030 | 0.0277 | 0.0488 | 0.0377 | 0.0791* | 0.0249 |
| Big5Open | 0.0834* | 0.0754* | 0.0842* | 0.0330 | 0.0047 | 0.0967* | 0.0376 |
| Big5Extra | -0.0056 | 0.0205 | 0.0565* | 0.0159 | 0.0070 | -0.0318 | 0.0273 |
| Big5Agree | 0.1157* | 0.0710* | 0.0968* | 0.0695* | 0.0598* | 0.1802* | 0.1337* |
| Big5Cons | 0.1289* | 0.0863* | 0.1045* | 0.0497 | 0.0336 | 0.1598* | 0.1132* |
| Big5Stab | 0.1167* | 0.1168* | 0.0477 | 0.0840* | 0.0698* | 0.1620* | 0.1336* |
| Wellbeing | 0.0795* | 0.0629* | 0.1215* | 0.0989* | 0.0558* | 0.1729* | 0.1054* |
| Truancy | 0.1601* | 0.0649* | 0.1063* | 0.1159* | 0.0993* | 0.1952* | 0.1396* |
| Pocket Money | 0.0046 | 0.0390 | 0.0074 | 0.0321 | 0.0439 | 0.0486 | 0.0407 |
| Pocket Money Spend | 0.0080 | 0.0828* | 0.0648* | 0.0589* | 0.0304 | 0.0324 | 0.0573* |
| Country | 0.0287 | 0.1677* | 0.0887* | -0.0086 | -0.0137 | 0.0359 | 0.1350* |
| Gender | 0.0082 | -0.0258 | 0.0057 | -0.0324 | 0.0233 | -0.0340 | 0.0041 |
| Age | -0.0913* | 0.0063 | -0.0267 | -0.0497* | -0.0267 | -0.1354* | -0.0823* |
| Single Parent | -0.0139 | -0.0437 | -0.0111 | -0.0504* | -0.0472 | 0.0008 | -0.0144 |

|  | Descriptive~8 | School Info | Advert~Media | Advert~Shops | Self Efficacy | Risk | Benefits |
| --- | --- | --- | --- | --- | --- | --- | --- |
| Desc_Classmates8 | 1.0000 |  |  |  |  |  |  |
| School Info | 0.0225 | 1.0000 |  |  |  |  |  |
| Advertising Media | -0.1111* | -0.0170 | 1.0000 |  |  |  |  |
| Advertising Shops | -0.0746* | -0.0405 | 0.2531* | 1.0000 |  |  |  |
| Self Efficacy | 0.2197* | 0.0677* | -0.2061* | -0.0738* | 1.0000 |  |  |
| Risk | 0.0002 | 0.0573* | -0.0358 | 0.0427 | 0.1887* | 1.0000 |  |
| Benefits | -0.0909* | -0.0537* | 0.1369* | 0.0688* | -0.1568* | 0.2724* | 1.0000 |
| PBC Quit | 0.0386 | -0.0554* | 0.0467 | -0.0543* | -0.0393 | -0.2469* | -0.0746* |
| PBC Avoid | 0.0508* | 0.0296 | -0.0793* | 0.0188 | 0.1907* | 0.0582* | -0.1612* |
| Attitude | 0.1172* | 0.0757* | -0.1384* | -0.0620* | 0.4032* | 0.2102* | -0.1593* |
| Knowledge | -0.0671* | 0.0569* | -0.0139 | 0.0974* | 0.0648* | 0.3030* | 0.0785* |
| Belong | -0.0706* | 0.0795* | -0.0114 | 0.0248 | 0.0147 | 0.1070* | 0.0386 |
| Negative | -0.0844* | -0.0046 | 0.0445 | 0.0787* | -0.0601* | 0.0753* | 0.0389 |
| Prosocial | 0.0439 | 0.0923* | -0.0506* | 0.0067 | 0.1160* | 0.1378* | -0.0876* |
| Big5Open | 0.0378 | -0.0232 | 0.0146 | 0.0223 | 0.1119* | 0.0767* | -0.0529 |
| Big5Extra | 0.0140 | 0.0135 | 0.0199 | 0.0572* | 0.0470 | 0.0336 | -0.0171 |
| Big5Agree | 0.0915* | 0.0676* | -0.0848* | -0.1140* | 0.1724* | 0.1104* | -0.0919* |
| Big5Cons | 0.1013* | 0.0396 | -0.0789* | -0.0997* | 0.1630* | 0.0739* | -0.0966* |
| Big5Stab | 0.1250* | 0.0572* | -0.0756* | -0.1124* | 0.1504* | -0.0234 | -0.0635* |
| Wellbeing | 0.0519* | 0.1652* | -0.0700* | -0.0930* | 0.2452* | 0.1253* | -0.1228* |
| Truancy | 0.1257* | 0.1144* | -0.1365* | -0.0609* | 0.2598* | 0.1409* | -0.1198* |
| Pocket Money | 0.0136 | -0.0417 | -0.0845* | 0.0356 | 0.0125 | 0.0098 | -0.0105 |
| Pocket Money Spend | 0.0164 | -0.0320 | -0.0273 | -0.0095 | 0.0331 | 0.0114 | -0.0487 |
| Country | 0.1268* | -0.1721* | 0.1066* | -0.1345* | -0.0824* | -0.2968* | 0.0430 |
| Gender | -0.0853* | -0.0514* | 0.0008 | -0.0189 | -0.0244 | 0.0605* | 0.0102 |
| Age | -0.0190 | -0.0796* | 0.0804* | 0.0454 | -0.1688* | -0.1278* | -0.0205 |
| Single Parent | 0.0449 | -0.0551* | 0.0584* | -0.0336 | -0.0806* | -0.1124* | 0.0024 |

|  | PBC Quit | PBC Avoid | Attitude | Knowledge | Belong | Negative | Prosocial |
| --- | --- | --- | --- | --- | --- | --- | --- |
| PBC Quit | 1.0000 |  |  |  |  |  |  |
| PBC Avoid | 0.3069* | 1.0000 |  |  |  |  |  |
| Attitude | -0.0166 | 0.1493* | 1.0000 |  |  |  |  |
| Knowledge | -0.1688* | 0.0436 | 0.1586* | 1.0000 |  |  |  |
| Belong | -0.0752* | 0.0389 | 0.0367 | 0.0738* | 1.0000 |  |  |
| Negative | -0.0889* | 0.0187 | -0.0479 | 0.0829* | 0.5563* | 1.0000 |  |
| Prosocial | -0.0907* | 0.1314* | 0.1462* | 0.1554* | 0.1928* | 0.1312* | 1.0000 |
| Big5Open | 0.0733* | 0.1052* | 0.1675* | 0.0972* | -0.0174 | -0.0088 | 0.3489* |
| Big5Extra | 0.0425 | 0.0637* | 0.0549 | 0.0713* | 0.0002 | -0.1616* | 0.2528* |
| Big5Agree | 0.0179 | 0.1204* | 0.2447* | 0.0632* | 0.0799* | -0.0006 | 0.4559* |
| Big5Cons | 0.0470 | 0.1144* | 0.2506* | 0.0869* | -0.0034 | -0.0412 | 0.3093* |
| Big5Stab | 0.0472 | 0.0155 | 0.1122* | -0.0596* | -0.1924* | -0.3793* | 0.0236 |
| Wellbeing | -0.0289 | 0.0934* | 0.2219* | 0.0547* | -0.0183 | -0.1586* | 0.1584* |
| Truancy | -0.0904* | 0.1101* | 0.1716* | 0.1151* | 0.0548* | 0.0296 | 0.1043* |
| Pocket Money | -0.0524* | 0.0177 | 0.0301 | 0.0464 | 0.0066 | 0.0388 | -0.0007 |
| Pocket Money Spend | 0.0006 | -0.0130 | 0.0821* | 0.0023 | 0.0326 | 0.0615* | 0.0439 |
| Country | 0.3273* | -0.1156* | -0.0518* | -0.2947* | -0.2607* | -0.2499* | -0.1814* |
| Gender | -0.0369 | -0.0184 | 0.0002 | 0.0306 | 0.0384 | 0.0942* | 0.1668* |
| Age | 0.0648* | -0.0237 | -0.1247* | -0.0781* | -0.0832* | -0.0597* | -0.0398 |
| Single Parent | 0.0948* | -0.0450 | -0.0484 | -0.0539* | -0.0791* | -0.0712* | -0.0317 |

|  | Big5Open | Big5Extra | Big5Agree | Big5Cons | Big5Stab | Wellbeing | Truancy |
| --- | --- | --- | --- | --- | --- | --- | --- |
| Big5Open | 1.0000 |  |  |  |  |  |  |
| Big5Extra | 0.4697* | 1.0000 |  |  |  |  |  |
| Big5Agree | 0.4023* | 0.3404* | 1.0000 |  |  |  |  |
| Big5Cons | 0.4123* | 0.3087* | 0.5649* | 1.0000 |  |  |  |
| Big5Stab | 0.0574* | 0.2608* | 0.3375* | 0.3709* | 1.0000 |  |  |
| Wellbeing | 0.0991* | 0.2008* | 0.2723* | 0.2691* | 0.3133* | 1.0000 |  |
| Truancy | 0.0316 | -0.0002 | 0.1612* | 0.1532* | 0.0566* | 0.1815* | 1.0000 |
| Pocket Money | -0.0382 | -0.0408 | 0.0075 | -0.0467 | -0.0255 | -0.0820* | 0.0689* |
| Pocket Money Spend | 0.0471 | -0.0231 | 0.0772* | 0.0457 | 0.0173 | 0.0279 | 0.1113* |
| Country | 0.2048* | 0.0960* | 0.0605* | 0.0906* | 0.1550* | -0.0892* | -0.2336* |
| Gender | 0.1337* | 0.0256 | 0.1022* | 0.0488 | -0.1953* | -0.0978* | 0.0315 |
| Age | 0.0346 | 0.0320 | -0.0929* | -0.0664* | -0.0117 | -0.1469* | -0.2203* |
| Single Parent | 0.0395 | 0.0437 | -0.0149 | 0.0138 | 0.0799* | -0.1082* | -0.1070* |

|  | Pocket~y | Pocket~d | Country | Gender | Age | Single~t |
| --- | --- | --- | --- | --- | --- | --- |
| Pocket Money | 1.0000 |  |  |  |  |  |
| Pocket Money Spend | 0.2350* | 1.0000 |  |  |  |  |
| Country | -0.0917* | -0.0289 | 1.0000 |  |  |  |
| Gender | 0.0017 | -0.0134 | -0.0307 | 1.0000 |  |  |
| Age | -0.0077 | -0.0553* | 0.1816* | -0.0967* | 1.0000 |  |
| Single Parent | 0.0021 | -0.0022 | 0.2270* | 0.0131 | 0.0789* | 1.0000 |

| Table 6: Variation inflation factor and tolerance scores | | | | |
| --- | --- | --- | --- | --- |
| Variable | VIF | SQRT VIF | Tolerance | R-Squared |
| **Socio-Environmental Factors** |  |  |  |  |
| Injunctive Norms |  |  |  |  |
| Important people | 1.30 | 1.14 | 0.7688 | 0.2312 |
| Mother | 1.41 | 1.19 | 0.7096 | 0.2904 |
| Father | 1.32 | 1.15 | 0.7590 | 0.2410 |
| Brother(s) | 1.31 | 1.15 | 0.7605 | 0.2395 |
| Sister(s) | 1.27 | 1.12 | 0.7904 | 0.2096 |
| Friends | 1.87 | 1.37 | 0.5345 | 0.4655 |
| Best friend | 1.83 | 1.35 | 0.5475 | 0.4525 |
| Descriptive Norms |  |  |  |  |
| Best friend | 1.32 | 1.15 | 0.7591 | 0.2409 |
| Mother | 1.33 | 1.15 | 0.7528 | 0.2472 |
| Father | 1.26 | 1.12 | 0.7947 | 0.2053 |
| Brother(s) | 1.13 | 1.06 | 0.8881 | 0.1119 |
| Sister(s) | 1.11 | 1.05 | 0.9040 | 0.0960 |
| Friends | 1.52 | 1.23 | 0.6582 | 0.3418 |
| Family | 1.43 | 1.20 | 0.6996 | 0.3004 |
| Classmates | 1.23 | 1.11 | 0.8105 | 0.1895 |
| School Smoking Information | 1.08 | 1.04 | 0.9256 | 0.0744 |
| Smoking in Media | 1.17 | 1.08 | 0.8559 | 0.1441 |
| Smoking in Shops | 1.16 | 1.08 | 0.8634 | 0.1366 |
| **Smoking-Related Cognitions** |  |  |  |  |
| Self-Efficacy | 1.46 | 1.21 | 0.6861 | 0.3139 |
| Perceived Risks | 1.51 | 1.23 | 0.6638 | 0.3362 |
| Perceived Benefits | 1.27 | 1.13 | 0.7895 | 0.2105 |
| PBC Quit | 1.34 | 1.16 | 0.7456 | 0.2544 |
| PBC Avoid | 1.28 | 1.13 | 0.7798 | 0.2202 |
| Attitude | 1.42 | 1.19 | 0.7040 | 0.2960 |
| Knowledge | 1.24 | 1.11 | 0.8051 | 0.1949 |
| **Psychosocial Factors** |  |  |  |  |
| Need to Belong | 1.62 | 1.27 | 0.6166 | 0.3834 |
| Fear of Negative Evaluation | 1.79 | 1.34 | 0.5597 | 0.4403 |
| Prosociality | 1.51 | 1.23 | 0.6630 | 0.3370 |
| Openness | 1.68 | 1.30 | 0.5944 | 0.4056 |
| Extraversion | 1.52 | 1.23 | 0.6567 | 0.3433 |
| Agreeableness | 1.93 | 1.39 | 0.5169 | 0.4831 |
| Conscientiousness | 1.77 | 1.33 | 0.5650 | 0.4350 |
| Stability | 1.61 | 1.27 | 0.6192 | 0.3808 |
| Wellbeing | 1.30 | 1.14 | 0.7670 | 0.2330 |
| Truancy | 1.25 | 1.12 | 0.7984 | 0.2016 |
| Pocket Money | 1.12 | 1.06 | 0.8967 | 0.1033 |
| Pocket Money Spending | 1.12 | 1.06 | 0.8893 | 0.1107 |

Figure 1: Receiver operating characteristic curve (both countries)


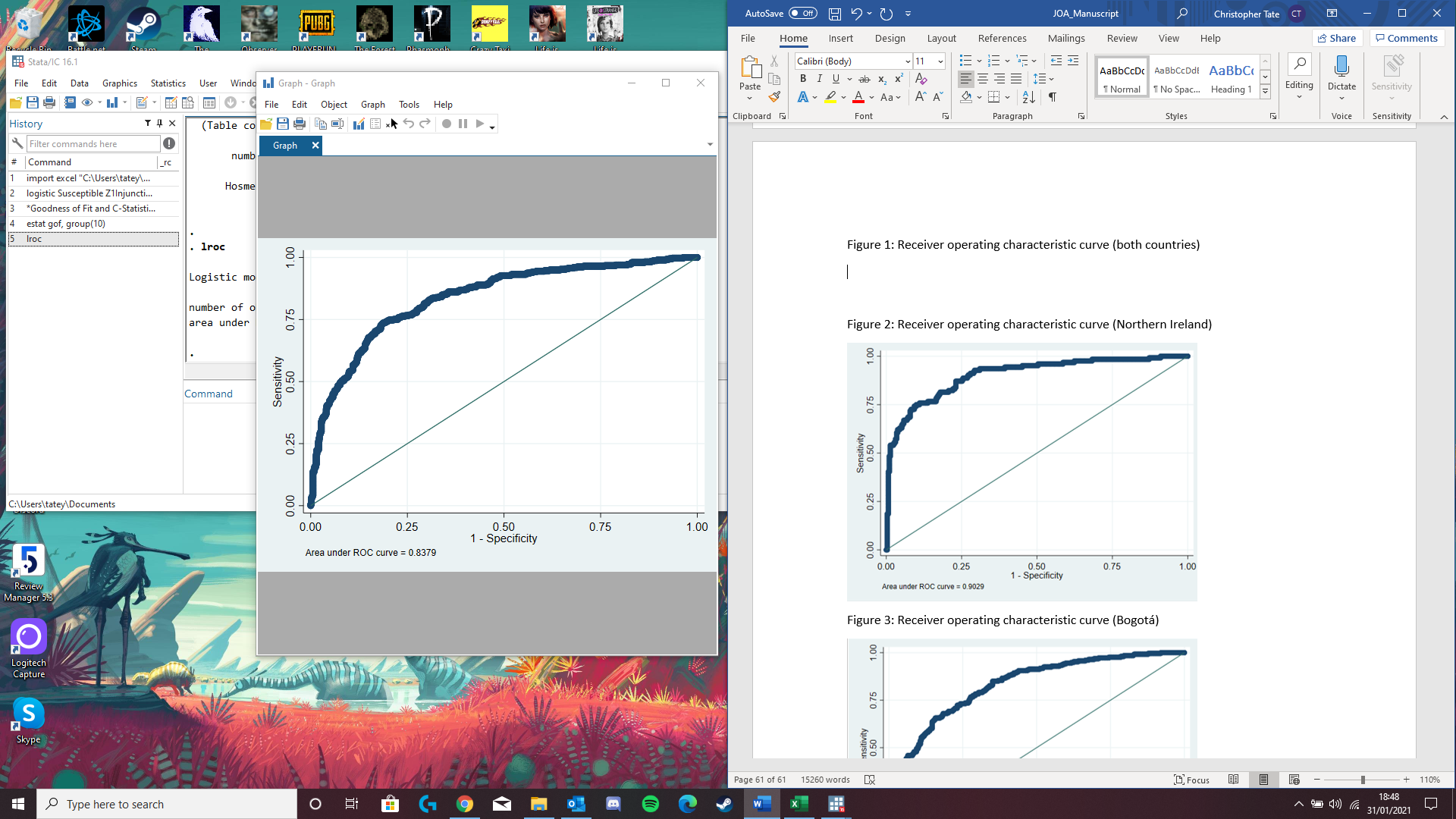


Figure 2: Receiver operating characteristic curve (Northern Ireland)


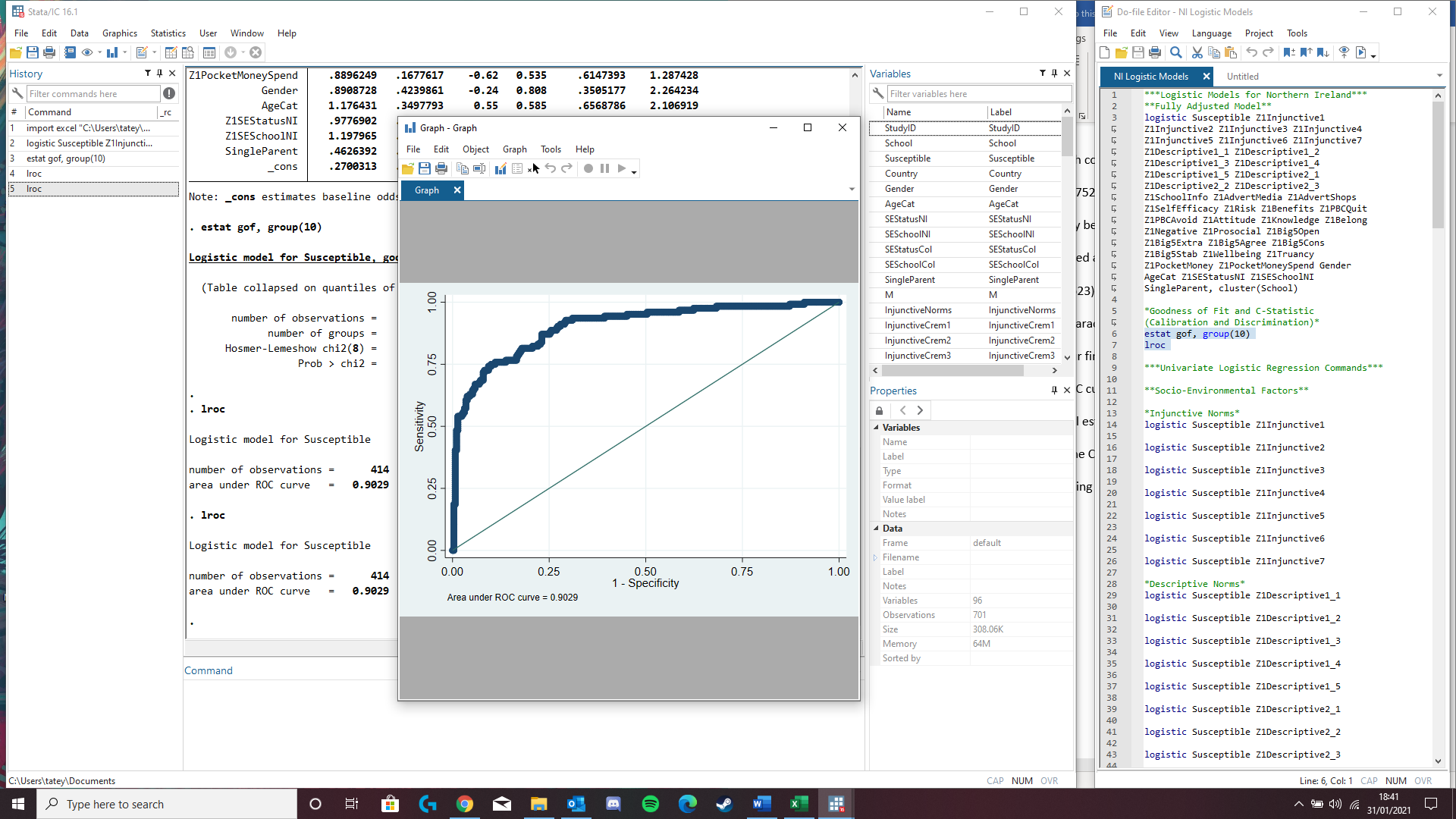


Figure 3: Receiver operating characteristic curve (Bogotá)


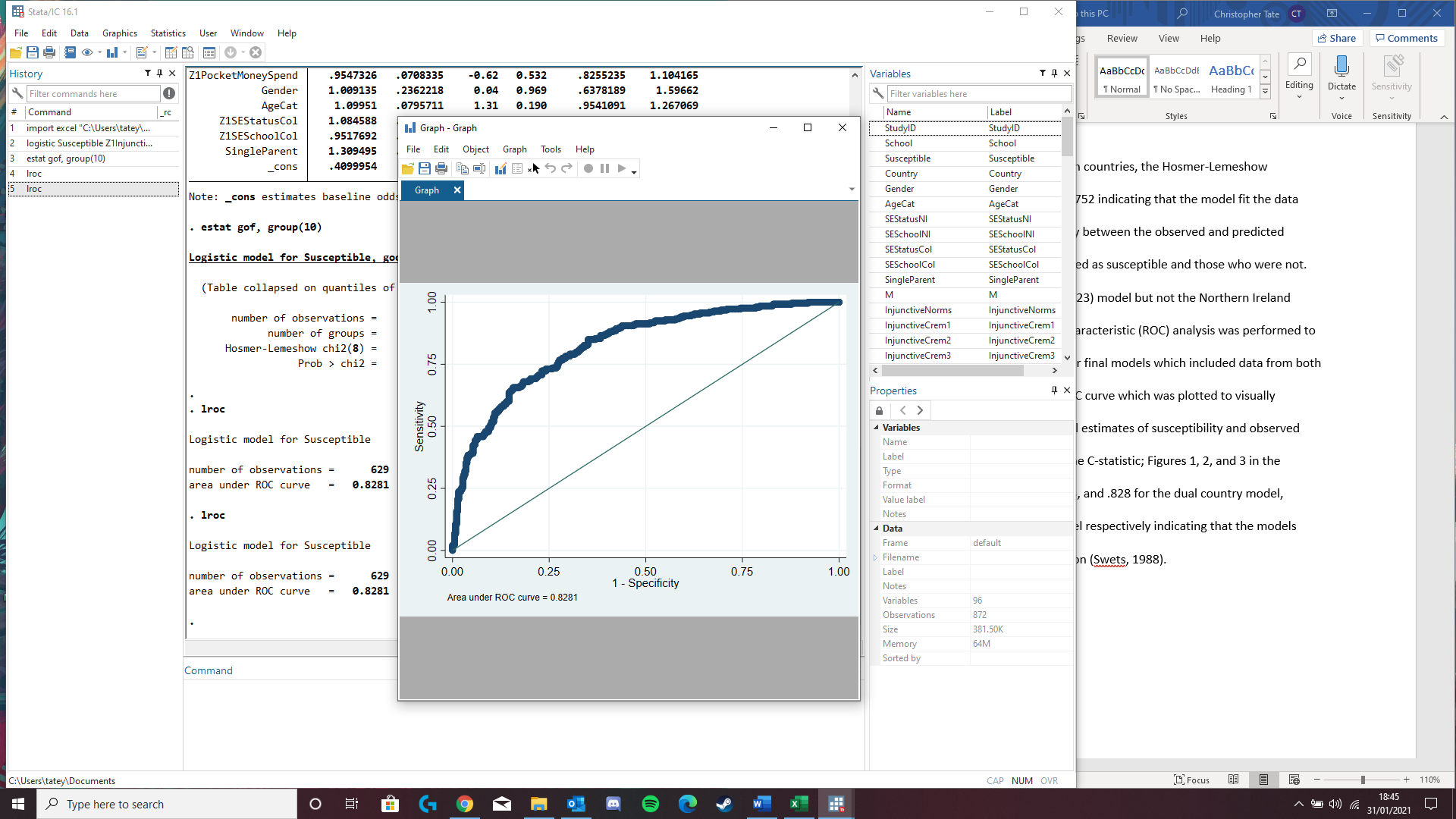

Supplement: Supplementary file 1 — Additional file 1. [file 12889_2021_12351_MOESM1_ESM.docx]
